# GSTRPCA: irregular tensor singular value decomposition for single-cell multi-omics data clustering

**DOI:** 10.1093/bib/bbae649

**Published:** 2024-12-16

**Authors:** Lubin Cui, Guiliang Guo, Michael K Ng, Quan Zou, Yushan Qiu

**Affiliations:** School of Mathematics and Statistics, Henan Normal University, Xinxiang 453007, China; School of Mathematics and Statistics, Henan Normal University, Xinxiang 453007, China; Department of Mathematics, Hong Kong Baptist University, Hong Kong 999077, China; Institute of Fundamental and Frontier Sciences, Electronic Science and Technology University, Chengdu 611731, China; School of Mathematical Sciences, Shenzhen University, Guangdong 518000, China

**Keywords:** single-cell multi-omics data, irregular tensor decomposition, weighted threshold, joint tensor

## Abstract

Single-cell multi-omics refers to the various types of biological data at the single-cell level. These data have enabled insight and resolution to cellular phenotypes, biological processes, and developmental stages. Current advances hold high potential for breakthroughs by integrating multiple different omics layers. However, singlecell multi-omics data usually have different feature dimensions and direct or indirect relationships. How to keep the data structure of these different data and extract hidden relationships is a major challenge for omics data integration, and effective integration models are urgently needed. In this paper, we propose an irregular tensor decomposition model (GSTRPCA) based on tensor robust principal component analysis (TRPCA). We developed a weighted threshold model for the decomposition of irregular tensor data by combining low-rank and sparsity constraints, which requires that the low-dimensional embeddings of the data remain lowrank and sparse. The major advantage of the GSTRPCA algorithm is its ability to keep the original data structure and explore hidden related features among omics data. For GSTRPCA, we also designed an effective algorithm that theoretically guarantees global convergence for the tensor decomposition. The computational experiments on irregular tensor datasets demonstrate that GSTRPCA significantly outperformed the state-of-the-art methods and hence confirm the superiority of GSTRPCA in clustering single-cell multiomics data. To our knowledge, this is the first tensor decomposition method for irregular tensor data to keep the data structure and hence improve the clustering performance for single-cell multi-omics data. GSTRPCA is a Matlabbased algorithm, and the code is available from https://github.com/GGL-B/GSTRPCA.

## Introduction

Single-cell multi-omics technology is a powerful approach for the simultaneous detection of multi-omics layers, including the genome, transcriptome, epigenome, metabolome, and proteome, in individual cells [1]. This integrative approach offers a comprehensive and precise understanding of cellular information, thereby facilitating deeper insights into cell function and phenotypic characteristics. CITE-seq (cellular indexing of transcriptomes and epitopes) is a commonly used method in single-cell multi-omics studies because it combines single-cell RNA sequencing (scRNA-seq) with protein epitome analysis to achieve high-resolution profiling of both RNA and proteins. However, the use of single-cell multi-omics technology has several challenges, including the sparsity of high-dimensional data, inherent dropout and noise, intricate non-linear structures, and the inherent heterogeneity observed among different omics datasets. Effectively overcoming these challenges, successfully integrating the diverse omics features, and accurately extracting cellular heterogeneity at the single-cell level are crucial prerequisites for downstream analysis of single-cell multi-omics data.

Several single-cell omics data integration methods have been developed to gain a more comprehensive understanding of the interactions and regulatory relationships among various biomolecules in organisms, thereby discovering important biological issues such as the pathogenesis of complex diseases, genetic differences between individuals, and cell types. Early integration methods include TotalVI (total variational inference) [2], a deep learning model that was designed to process histological datasets. SCMDC (single-cell multi-omics data clustering) [3] uses advanced clustering algorithms and machine learning techniques to analyze and process integrated multi-omics data, thereby exploring the biological complexity at the single-cell level. BREM-SC (Bayesian random effects mixture model) [4], an advanced technology that combines Bayesian statistics and representation learning, models and analyzes single-cell data, and provides powerful tools and methods to uncover biological complexity at the single-cell level. scMNMF (single-cell multi-omics clustering based on non-negative matrix factorization) [5] is a joint learning method that integrates dimensionality reduction and cell clustering analysis on single-cell multi-omics data using non-negative matrix factorization.

In addition, single-cell multi-omics data can be naturally characterized and analyzed as tensor data [6–8], whereby each single-cell omics dataset can be viewed as a regular tensor. Multi-omics data composed of different types and dimensions of single-cell omics data form irregular tensors that define potential connections between cells and genes from various perspectives. Extensive explorations have been conducted into the application of structured tensors in biology, extending robust principal component analysis (RPCA) [9] to conventional tensor data to develop tensor robust principal component analysis (TRPCA) [10]. TRPCA efficiently manages high-dimensional tensor data and extracts information from structured tensor data in biostatistics [11]. By representing protein interaction data as a third-order tensor, methods such as CP decomposition [12] and Tucker decomposition [13] can be used to discover latent structural insights, such as key interaction patterns or the composition of protein complexes. These decomposition methods effectively manage high-dimensional tensor data by handling outliers and missing values within datasets. Although robust methods for identifying differentially expressed genes in biological contexts are available, their application is confined to regular tensor omics data. Current approaches often use zero-padding to convert irregular tensors into structured tensors for processing, leading to issues such as high data redundancy and incomplete extraction of internal information structures, which adversely affect downstream analyses. Therefore, developing effective models and algorithms to handle irregular tensor multi-omics data is a pressing need.

Considering the aforementioned limitations and inspirations, we focused on the analysis of single-cell multi-omics data, with particular emphasis on irregular tensor data structures. We propose an irregular tensor singular value decomposition method (GSTRPCA) based on GSVD and weighted thresholding.Unlike traditional approaches,GSTRPCA does not require zero-padding of the original data, ensuring accurate recovery of low-rank components. We used weighted thresholding algorithms and techniques based on nuclear norms to effectively mine information from single-cell multi-omics data. We also conducted theoretical convergence analysis on the algorithm, and compared the iterative error curves between GSTRPCA and the competing algorithms to confirm the superior convergence rate of our method. In the clustering performance experiments, we combined irregular low-rank tensors and irregular sparse tensors for clustering. The results demonstrate that this approach outperformed traditional methods.

## Methods

###  

#### Tensor robust principal component analysis model

The regular tensor $\mathcal{X}\in \mathbb{R}^{N\times M\times K} $ is processed by TRPCA. The original data are decomposed into low-rank tensors $\mathcal{L}\in \mathbb{R}^{N\times M\times K} $ and sparse tensor $\mathcal{E}\in \mathbb{R}^{N\times M\times K }$, which can be approximated as the sum of low-rank tensors and sparse tensors: 


(2.1) \begin{align*}& \begin{aligned} \mathcal{X}\approx \mathcal{L}+\mathcal{E},\\ \end{aligned}\end{align*}

where $\mathcal{L}$ represents the reconstruction of the original data in low-rank space and $\mathcal{E}$ represents the reconstruction of the original data in sparse space. The general form of the tensor low-rank sparse decomposition model is formulated as: 


(2.2) \begin{align*}& \begin{aligned} \min_{\mathcal{L},\mathcal{E}}\;\; &[{Trank(L)+\lambda \left \| \mathcal{E} \right \|_{0}} ],\\ s.t. \;\; &\mathcal{X}=\mathcal{L}+\mathcal{\mathcal{E}}. \end{aligned}\end{align*}

Tensors $\mathcal{L}$ and $\mathcal{E}$ that satisfy the condition $\mathcal{X}=\mathcal{L}+\mathcal{E}$ are calculated to minimize the objective functions $Trank(\mathcal{L})+\lambda \left \| \mathcal{E} \right \|_{0}$, where $Trank(\mathcal{L})$ is the rank of $\mathcal{L}$, $\left \| \mathcal{E} \right \|_{0}$ is the $\left \| \mathcal{L} \right \|_{0}$ norm on $\mathcal{E}$, and $\lambda> 0$ is a constant.

Because the solution of Equation (2.2) is an NP-complete problem, the usual strategy is to transform Equation (2.2) using $\left \| \mathcal{L} \right \|_{*}$ and replace $\left \| \mathcal{E} \right \|_{0}$ with $\left \| \mathcal{E} \right \|_{1}$. Then the objective function of TRPCA becomes 


(2.3) \begin{align*}& \begin{aligned} \min_{\mathcal{L},\mathcal{E}}\;\; &{\left \| \mathcal{L} \right \|_{*}+\lambda \left \| \mathcal{E} \right \|_{1}} \\ s.t. \;\; &\mathcal{X}=\mathcal{L}+\mathcal{E}, \end{aligned}\end{align*}

where $\left \| \cdot \right \|_{*}$ represents the tensor kernel norm and $\lambda $ is the degree of punishment that affects sparse structures.

###  

#### Decomposition method for irregular tensor (GSTRPCA)

The single-cell multi-omics data are irregular tensor data $\mathcal{X}\in \mathbb{R}^{(N_{1},N_{2}, \dots ,N_{K})\times M\times K }$, where $\mathcal{X}$ represents a matrix composed of $N1 * M$, $N2 * M, \dots , N_{K} * M$ matrices. For $\mathcal{X}$, the irregular tensor forms are all assumed to be irregular three-dimensional array forms, and the matrix size for constructing tensors is different in the rows and the same in the columns. The irregular tensor dimensions are referred to as $\mathbb{R}^{N_{i}\times M\times K }$.

Based on the TRPCA model (2.3), $\mathcal{L}\in \mathbb{R}^{N_{i}\times M\times K }$ represents an irregular low-rank tensor and $\mathcal{E} \in \mathbb{R}^{N_{i}\times M\times K }$ represents an irregular sparse tensor. We aimed to decompose the original irregular tensor into an irregular low-rank tensor and an irregular sparse tensor. Then, by combining the low-rank and sparse constraints, the global subspace and local geometric structure of the data can be captured by the reconstruction tensor while maintaining the low-rank and sparsity constraints in the low-dimensional embedding of the data to ensure overall optimality. Furthermore, by integrating the global subspace and local geometric structure into a unified framework using low-rank and sparse embeddings, optimal clustering performance can be ensured at all times.

###  

#### Optimization of the algorithm

To solve $\left \| \mathcal{L} \right \|_{*}$, GSTRPCA uses GSVD instead of SVD to solve the kernel norm. (Specific details of the GSVD algorithm are given in the supplementary file.) For the irregular tensor data, GSVD can decompose it directly, whereas SVD requires the original data to be filled a regular tensor before decomposition, which destroys the structure of the data and makes it sparser. Thus, GSTRPCA, which based on GSVD, has greater potential to capture data structures and improve single-cell multi-omics clustering.

It is difficult to directly find the optimal solution of the objective function for $\mathcal{E}_{1}$ norm as the regularization of sparse terms. Under the alternating direction method of multipliers (ADMM) framework, an augmented Lagrangian function is introduced to eliminate equality constraints. Equation (2.3) can be rewritten as augmented Lagrangian functions: 


(2.4) \begin{align*}& \begin{aligned} P(\mathcal{L},\mathcal{E},\mathcal{Y},\mu )&=\left \| \mathcal{L} \right \|_{*}+\lambda \left \| \mathcal{E} \right \|_{1} +<\mathcal{Y},\mathcal{L}+\mathcal{E} -\mathcal{X}>\\ &\quad +\frac{\mu }{2}\left \| \mathcal{L} +\mathcal{E} -\mathcal{X} \right \|_{F}^{2}, \end{aligned}\end{align*}

where $\mathcal{Y}$ is the dual variable, and $\mu $ is the introduced equilibrium parameter.

Then under the ADMM framework, the preliminary updating formulas for $\mathcal{L}^{k+1}$ and $\mathcal{E}^{k+1}$ are: 


(2.5) \begin{align*} & \begin{aligned} \mathcal{L}^{k+1}&=\mathop{\arg\min}\limits_{\mathcal{L}} P(\mathcal{L},\mathcal{E}^{k},\mathcal{Y}^{k},\mu^{k})\\ &=\mathop{\arg\min}\limits_{\mathcal{L}}\left \| \mathcal{L} \right \|_{*} +\frac{\mu^{k}}{2}\left \| \mathcal{L} +\mathcal{E}^{k} -\mathcal{X}+\frac{\mathcal{Y}^{k}}{\mu^{k}} \right \|_{F}^{2}.\quad\ \ \end{aligned} \end{align*}

(2.6) \begin{align*} & \begin{aligned} \mathcal{E}^{k+1}&=\mathop{\arg\min}\limits_{\mathcal{E}} P(\mathcal{L}^{k+1},\mathcal{E},\mathcal{Y}^{k},\mu^{k})\\ &=\mathop{\arg\min}\limits_{\mathcal{E}}\lambda \left \| \mathcal{E} \right \|_{1} +\frac{\mu^{k}}{2} \left \| \mathcal{L}^{k+1}+\mathcal{E} -\mathcal{X}+\frac{\mathcal{Y}^{k}}{\mu^{k}} \right\|_{F}^{2}. \end{aligned}\end{align*}

Details of how to solve irregular low-rank tensors $\mathcal{L}^{k+1}$ and irregular sparse tensors $\mathcal{E}^{k+1}$ are given in the supplementary file. Prove of the global theoretical convergence of the Algorithm 1 is also provided in the supplementary file. The run-time efficiency of GSTRPCA was consistently more than that of the other methods on different datasets (Supplementary Table 2). The framework of the algorithm is summarized in Algorithm 1.


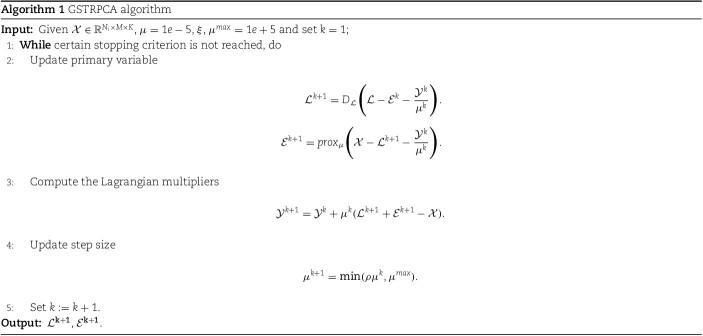


Irregular tensor decomposition was performed on simulated and real datasets to decompose them into low-rank and sparse irregular tensors. Then the tensor $\mathcal{X}^\ast $ was reconstructed using the $\mathcal{L}$ and $\mathcal{E}$. Finally, we conducted cell clustering experiments on $\mathcal{X}^\ast $ using the method proposed in [14] and the clustering performance was further evaluated.

**Figure 1:**
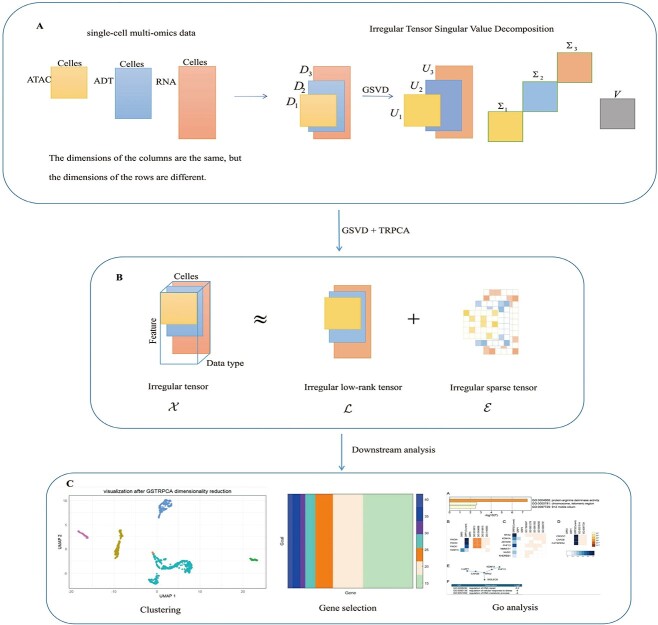
Comprehensive overview of GSTRPCA. (A) Input single-cell multi-omics data types and construct them as irregular tensor data for the generalized singular value decomposition of irregular tensors. (B) Decompose the irregular tensor data into an irregular low-rank tensor and a sparse tensor, which is more complex than a straightforward low-rank approximation, as it incorporates considerations of sparsity. In the depicted irregular sparse tensor, the majority of the elements are zero. (C) We conducted a comprehensive analysis of single-cell multi-omics data, employing UMAP (uniform manifold approximation and projection) for the visualization of cellular clustering. Additionally, we performed gene selection and GO enrichment analysis to identify biological processes and functions associated with the selected genes.

Here, we developed a novel method (GSTRPCA) based on TRPCA to improve the weighted threshold for the decomposition of irregular tensor data (Fig. 1). On the Fig. 1A left side, the input single-cell multi-omics data comprises three types: ATAC, RNA, and ADT. While the dimensions of the rows differ among these datasets, they share the same number of columns. On the right side, an irregular tensor singular value decomposition (ITSVD) is performed. The input data $D_{i}\in \mathbb{R}^{M_{i}\times N }$ is processed through GSVD, which first constructs a common subspace S for the data matrix, followed by the computation of its decomposition. After decomposition, we obtain $U_{i}\in \mathbb{R}^{M_{i}\times N }$, $\Sigma _{i}\in \mathbb{R}^{M_{i}\times N }$, $V\in \mathbb{R}^{N\times N }$, where $\Sigma $ is a diagonal matrix with the singular values as its elements. As shown in Figure 1B, we leverage and enhance TRPCA and GSVD to decompose single-cell multi-omics data. Building on the foundation of TRPCA, we implement an improved threshold-based weighted algorithm to decompose the irregular tensor $\mathcal{X}\in \mathbb{R}^{N_{i}\times M\times K }$ into an irregular low-rank tensor $\mathcal{L}$ and $\mathcal{E}$. As shown in Figure 1C, we conducted subsequent downstream analyses on the results. The UMAP clustering visualization of the single-cell multi-omics data reveals a pronounced clustering effect using the GSTRPCA method. We performed gene selection on all resulting genes, ultimately identifying the top 50 genes and generating a heatmap to illustrate their expression. Additionally, we conducted Gene Ontology (GO) enrichment analysis on these selected genes.

## Experimental results

###  

#### Datasets

We evaluated the clustering performance of the GSTRPCA using five irregular single-cell multi-omics tensor datasets with different matrix sizes (two were simulated and three were real datasets). Details of these datasets are summarized in Table 1.

**Table 1:** Single-cell multi-omics datasets used in this study

Dataset	Cell	RNA	ADT	ATAC	Type	Refs
Sim1	529	2000		5000	5	[15]
Sim2	249	2500		5000	5	[15]
scGEM	177	34	27		5	[16]
Specter	3762	33538	49		16	[17]
10X_inhouse	1182	33538	10		6	[4]

- Simulated datasets We used two single-cell multi-omics datasets (Sim1 and Sim2), each containing gene expression omics and epigenetics omics data, that were generated previously [15]. Sim1 contains 529 cells with 5 cell types, and Sim2 contains 249 cells with 5 cell types.
- scGEM dataset The scGEM dataset contains 177 cells with 5 cell types. The data were extracted from [16].
- Specter dataset The Specter dataset contains 3762 cells with 16 cell types. The true labels are from Specter [17].
- 10X_inhouse dataset The 10X_inhouse dataset is a in_house CITE-seq dataset of human peripheral blood mononuclear cells from a healthy donor under institutional review board approval from the University of Pittsburgh, generated by [4]. This dataset contains 1182 cells with 6 cell types.

###  

#### Data preprocessing

The main purpose of data preprocessing is to eliminate low-quality and non-expressed feature gene to enhance the accuracy and reliability of subsequent analyses. The preprocessing steps for the irregular tensor $\mathcal{X}\in \mathbb{R}^{(N_{1},N_{2},...,N_{K})\times M\times K }$ in this paper are as follows:

1. Remove unexpressed genes. For the single-cell multi-omics data
  \begin{align*} &\mathcal{X}\in \mathbb{R}^{(N_{1},N_{2},\cdots,N_{K})\times M\times K },\end{align*}
  feature gene are filtered based on gene expression levels, chromatin accessibility data, and protein expression across individual cells. Specifically, we exclude genes that exhibit zero expression in all omics cells.
2. Remove low expressed genes. We filter out feature genes detected in only a small subset of cells. To achieve this, we calculate the proportion of each characterized gene that is expressed in the cell: genes are recorded as 1 when they are expressed in the cell and 0 when they are not expressed. We select genes that have expression in over 80% of the total cells to reduce noise and computational burden.

After that, we can get the integrated irregular tensor. Table 2 shows the detailed information of the five datasets. Taking the real dataset $10X{\_ }inhouse$ as an example, before data preprocessing this dataset consists of 1182 cells with an RNA gene expression number of 33538 and an ADT expression of 10. After data preprocessing, the data consists of a 10 $\times $ 1182 matrix and 10362 $\times $ 1182 matrix-type data. These data consist of matrices representing two different dimensions in the rows, with a sample cell number of 1182 and a total of six cell types, corresponding to irregular tensor data of the form $\mathcal{X}\in \mathbb{R}^{(10,10362)\times 1182\times 2}$.

**Table 2:** Irregular tensors for single-cell multi-omics datasets

Dataset	Cells	Features	Type	Irregular tensor data
Sim1	529	[1717;2296]	5	(1717,2296)$\times $529$\times $2
Sim2	249	[2088;4564]	5	(2088,4564)$\times $249$\times $2
scGEM	177	[27;32]	5	(27,32)$\times $177$\times $2
Specter	3762	[49;1000]	16	(49,1000)$\times $3762$\times $2
10X_inhouse	1182	[10;10362]	6	(10,10362)$\times $1182$\times $2

###  

#### Clustering performance

We compared our proposed GSTRPCA method with eight advanced methods; five methods based on tensor decomposition and three typical single-cell multi-omics data clustering methods. The five typical tensor decomposition clustering methods are:

- t-TRPCA (tensor robust principal component analysis) [18], which has been extended to the TRPCA model and widely applied to image processing and bioinformatics.
- LRTV (low-rank matrix total-variation-regularized) [19], which can be used to evaluate the informativeness of features and help select the most relevant and effective features for model construction in data mining and machine learning.
- LRTD (low-rank tensor decomposition) [20], which is widely applied method in data analysis and machine learning where it is used to decompose and reduce the dimensionality of high-order tensors. In bioinformatics, LRTD has been applied to analyze genomic data where it aided the discovery of intricate patterns and relationships among organisms.
- TT-TRPCA (tensor train for tensor robust principal component analysis) [21], which considers a new model for TRPCA based on tensor train rank that aims to recover a low-rank tensor corrupted by sparse noise.
- LLRGTV (local low-rank matrix recovery and global total variation) [22], which is commonly used for dimensionality reduction, signal processing, and image restoration where it has achieved excellent results.

The three single-cell multi-omics data clustering methods are:

- SCMDC (single-cell multi-omics data clustering) [3], which is a deep learning model that was designed to process different histological datasets. SCMDC extracts complex data features from raw data using deep learning techniques. After the integration is completed, clustering analysis is carried out using the jointly obtained potential features.
- BREM-SC (Bayesian random effects mixture model) [4], which is a computational model designed for the analysis of scRNA-seq data that effectively solves the problems of dimensionality reduction, and visualization and clustering of cellular gene expression profiles.
- TotalVI [2], which is a probabilistic deep learning framework that was specifically designed to address challenges in scRNA-seq analysis, such as dealing with high-dimensional data and handling missing values.

The clustering accuracy of GSTRPCA was compared with the clustering accuracy of the eight methods on the five datasets (Table 1) using four evaluation metrics, accuracy (ACC), normalized information (NMI), adjusted rand index (ARI), and adjusted mutual information (AMI), to evaluate the clustering performances.

**Figure 2:**
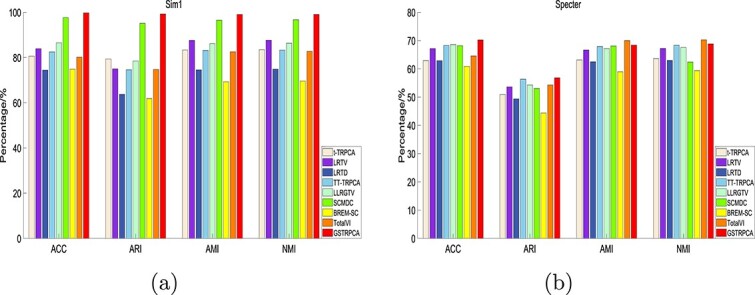
Evaluation metrics for the clustering performance of GSTRPCA and eight competing methods on (a) Sim1 and (b) Specter datasets.

These metrics measure the clustering performance of the method from different perspectives; high values indicating good clustering performance. On the Sim1 dataset, the performance of GSTRPCA was consistently better than those of the other eight methods in all four metrics (Fig. 2). On the Specter dataset, the performance of GSTRPCA was also consistently better than those of the other eight methods in ACC and ARI. In AMI and NMI, GSTRPCA ranked second and its clustering performance was comparable to that of TotalVI. And the clustering results for the other three datasets are given in Supplementary Fig. 8, and confirm the superior performance of GSTRPCA. On the 10X_inhouse dataset, the performance of GSTRPCA slightly lags that of SCMDC, probably because SCMDC can adapt to datasets with more cell types. Although SCMDC can extract complex structural features, the results show that the clustering performance of SCMDC on most of the datasets was not as good as that of GSTRPCA. The poorer performance of SCMDC may be explained by the inability of SCMDC to effectively analyze single-cell datasets for different molecular levels and to deal with their interrelationships.

Overall, the ARI, AMI, NMI, and ACC evaluation metrics ranked GSTRPCA in the top two for the five datasets and GSTRPCA performed better that the other methods tested. The superior performance of our method may lie in the weighted threshold processing used in the subsequent cell clustering process. We conclude that our proposed GSTRPCA method is effective and robust for improving the clustering of single-cell multi-omics data.

**Figure 3:**
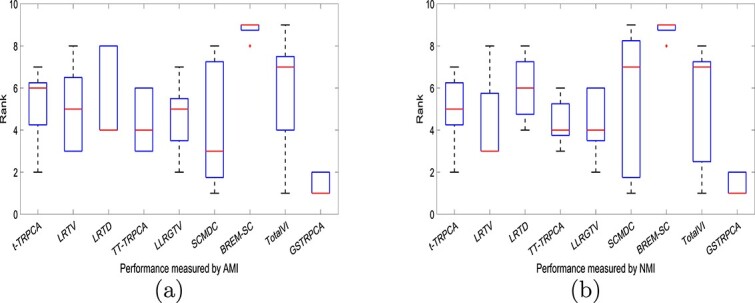
(a) Boxplots of GSTRPCA and other competing methods in terms of AMI on five datasets. (b) Boxplots of GSTRPCA and other competing methods in terms of NMI on five datasets. The minimum value, lower quartile, median (red line), upper quartile, and maximum value are shown. The length of a box (the interquartile range) indicates the stability of the method; the bigger the range the more unstable the method is, and a high ranking in the relative stability indicates a better method.

To visualize the clustering performance more intuitively, we used boxplots to summarize the ranking results of GSTRPCA and the competing methods (Fig. 3). The clustering results for each of the methods varied for each dataset. Outliers represent performance results that were not within the overall interval. Figure 3 shows that GSTRPCA outperformed the other competing methods on single-cell multi-omics datasets. And the boxplots for ACC and ARI are given in the Supplementary Fig. 9, which confirms the stability and superiority of our proposed method.

**Figure 4:**
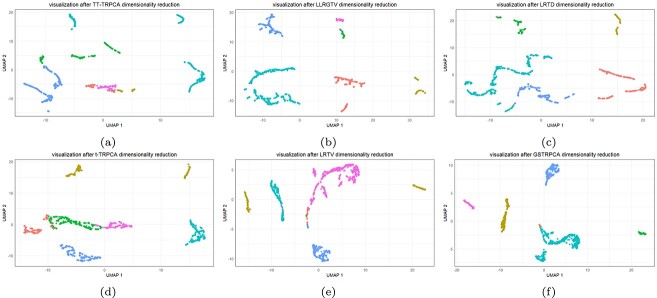
Visualization of cell clusters after dimensionality reduction for different methods on the 10X_inhouse dataset. (a) TT-TRPCA, (b) LRTD, (c) LLRGTV, (d) t-TRPCA, (e) LRTV, and (f) GSTRPCA.

We also used UMAP (uniform manifold approximation and projection ) to visualize the clustering performance of GSTRPCA and the other competing methods. Taking the 10X_inhouse dataset as an example, the results show that the different cell types were effectively separated by GSTRPCA (Fig. 4f), whereas all the cell types were not separated well by the other methods (Fig. 4a–e). Similar results were obtained for the other datasets (Supplementary Figs 4–7). Together, the results demonstrate that the GSTRPCA model was superior to the other methods tested and that it improved the cell clustering.

###  

#### Effect of irregular tensor decomposition

**Figure 5:**
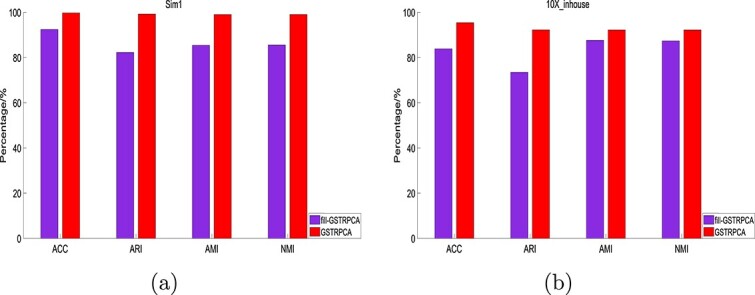
Clustering performances of the GSTRPCA decomposition of irregular tensor and fill-GSTRPCA decomposition of regular tensor on the (a) Sim1 and (b) 10X_inhouse datasets.

To investigate the effect of irregular tensor decomposition, we compared GSTRPCA for irregular tensor and the regular tensor constructed based on irregular tensor using zero-padding. We called the decomposition method for regular tensor fill-GSTRPCA, which implies that applying GSTRPCA to the regular tensor populated using zeros based on irregular tensor. We compared the clustering performance of these two methods on five datasets. The clustering performance of GSTRPCA was consistently better than that of fill-GSTRPCA on the Sim1 and 10X_inhouse datasets (Fig. 5). The clustering performances on the other datasets also show that GSTRPCA GSTRPCA was better than fill-GSTRPCA (Supplementary Fig. 10). These results may be because fill-GSTRPCA affects its own data structure, making the data sparser and destroying the geometric structure of the feature tensor. Therefore, to further develop GSTRPCA it is significantly important that the irregular tensor keeps the geometric structure and hence helps to improve the clustering for single-cell multi-omics data.

**Figure 6:**
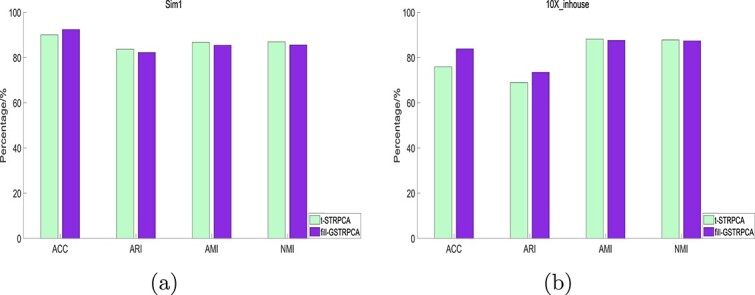
Clustering performances of fill-GSTRPCA and t-STRPCA decomposition methods on the (a) Sim1 and (b) 10X_inhouse datasets.

###  

#### Effect of GSVD decomposition

We also determined whether GSVD enhanced the clustering performance by comparing fill-GSTRPCA with weighted threshold decomposition based on T-SVD under zero-filling structure. We called the T-SVD decomposition method t-STRPCA.

On Sim1 and 10X_inhouse datasets, the clustering performance of fill-GSTRPCA was better than t-STRPCA in most of the comparisons (Fig. 6). The clustering performance results on different datasets are given in Supplementary Fig. 11, which also shows that fill-GSTRPCA slightly outperformed t-STRPCA. For filled sparse regular tensor data, t-STRPCA failed to effectively leverage the sparse properties. Inversion of sparse matrices during computation can lead to numerical stability issues, resulting in decreased accuracy of the decomposition results. In tensor decomposition, fill-GSTRPCA leverages information from different data modes to reduce data dimensionality and enhance efficiency in data analysis.

The overall evaluation results of GSTRPCA and the competing methods on different datasets are given in Table 3. The results show that GSTRPCA outperformed the other methods for various measures and demonstrate the superiority of decomposition on irregular tensor and GSVD.

**Table 3:** Evaluation metrics for GSTRPCA and the competing methods

Dataset	Evaluation indicators	Original	t-STRPCA	fill-GSTRPCA	GSTRPCA
Sim1	ACC	81.08	90.07	92.44	**99.76**
	ARI	71.12	83.69	82.27	**99.28**
	AMI	83.20	86.78	85.42	**99.06**
	NMI	83.38	86.96	85.57	**99.08**
Sim2	ACC	79.39	83.00	87.94	**94.98**
	ARI	74.27	74.14	74.76	**88.67**
	AMI	84.51	66.67	86.51	**91.35**
	NMI	84.50	67.24	86.70	**91.57**
scGEM	ACC	85.71	87.14	89.02	**92.86**
	ARI	69.86	71.09	75.31	**82.76**
	AMI	71.14	72.90	76.05	**83.86**
	NMI	72.23	73.92	76.95	**84.47**
Specter	ACC	62.94	67.84	69.11	**70.24**
	ARI	50.80	56.78	53.29	**56.83**
	AMI	62.83	67.73	66.83	**68.37**
	NMI	63.33	68.14	67.31	**68.83**
10X_inhouse	ACC	85.79	75.93	83.88	**95.44**
	ARI	74.97	68.96	73.50	**92.28**
	AMI	86.60	88.19	87.65	**92.25**
	NMI	86.41	87.84	87.40	**92.24**

## Downstream analysis

**Figure 7:**
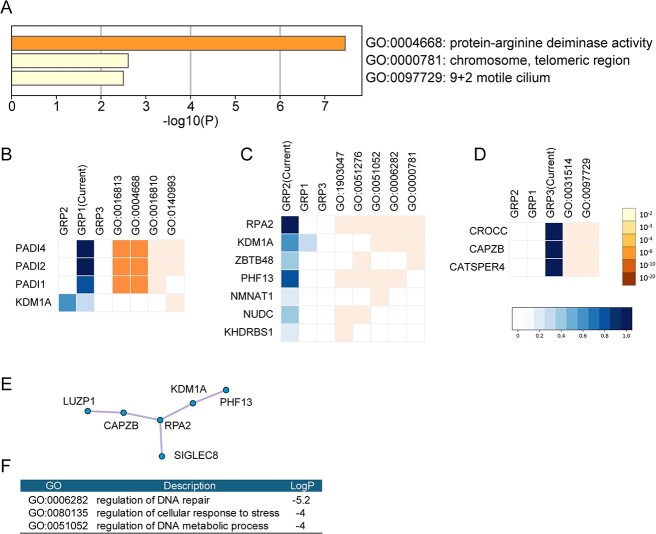
Enrichment analysis of 50 differentially expressed marker genes using Metascape. (A) Enriched GO terms under the biological process category. The colors are based on the *P*-values. (B–D) Heatmaps of genes associated with the three best-scoring GO groups, GRP1 (B), GRP2 (C), and GRP3 (D). The blue heatmaps (left) represent genes across groups. The darkness of the blue indicates the proportion of GO terms in a group associated with the gene. The orange heatmaps (right) represent genes across terms in the activated group. The darkness of the orange indicates the *P*-value of the GO term. (E) Protein–protein interaction network identified in the marker genes. (F)The three best-scoring GO terms by *P*-value.

We conducted downstream analysis of the GSTRPCA results and identified 50 differentially expressed information genes from the Specter dataset. Details of the 50 marker genes are listed in Supplementary Table 3. We conducted a GO enrichment analysis to predict the biological properties of the 50 genes using Metascape (https://metascape.org) (Fig. 7).

The functional enrichment analysis assigned GO terms under the biological processes, cellular component, and molecular function GO categories. The entire genome was used as the background for identifying enriched terms with *P*-values < 0.01. Terms with a minimum count of 3, and enrichment factor > 1.5 were collected and clustered based on their membership similarities. A protein–protein interaction network was constructed using the STRING, BioGrid, OmniPath, and InWeb_IM databases. Only physical interactions in STRING (physical score > 0.132) and BioGrid were considered.

Four marker genes associated with protein-arginine deiminase activity are shown in Fig. 7B, seven genes identified as components of the telomeric region on chromosomes are shown in Fig. 7C, and three genes identified as components of motile cilia are shown in Fig. 7D. A protein–protein interaction enrichment analysis predicted interactions among LUZP1, CAPZB, RPA2, KDM1A, and PHF13 (Fig. 7E). These genes are associated with DNA repair, cellular response to stress, and DNA metabolic processes (Fig. 7F).

Recent research suggests that the marker genes are primarily involved in crucial biological processes, such as protein arginine deaminate activity, DNA damage response, regulation of telomere stability, and ciliary motility. PADI2 and PADI4 have been implicated in neutrophil extracellular trap formation, which is linked to tumor metastasis and immune evasion as well as autoimmune diseases and cancers [23], insight into neutrophil extracellular traps [24], and prostate cancer cells [25]. RPA2 and KDM1A regulate the expression of immune-related genes to impact immune cell activation and function [26–28]. ZBTB48 modulates immune cell lifespan and function by preserving telomere integrity and genomic stability [29]. CATSPER4 influences intracellular calcium levels to affect T cell and natural killer cell activation and function [30, 31]. CAPZB and CROCC govern dynamic changes in cilia and cellular cytoskeleton to influence immune cell migration as well as the formation of immune synapses [32, 33]. Together, these findings suggest that the identified genes are significantly involved in modulating the immune response to tumors.

## Conclusions

Cell clustering is an important and rapidly developing direction in single-cell research. Clustering combines different types of single-cell data, such as gene expression, protein expression, and chromatin states, to comprehensively classify and cluster individual cells. This integrated analysis provides comprehensive cell type identification and functional interpretation, which aids in understanding the complex biological characteristics of cells. Single-cell multi-omics data form an irregular tensor, but, so far, no effective tensor decomposition methods have been developed to keep the original data structure and identify hidden related features among omics data.

In this study, we propose a novel method (GSTRPCA) based on TRPCA to improve the weighted threshold for the decomposition of irregular tensor data. For datasets that contained two types of genomic data, we first compared GSTRPCA with traditional tensor methods. Unlike previous rule tensor processing kernel norm methods, we performed generalized singular value decomposition on irregular tensors, and threshold processing on irregular low-rank tensors to better approximate the rank function and extract more unit structure information. We also validated the effectiveness and superiority of decomposition based on the irregular tensor. Furthermore, we compared the performance of GSTRPCA and t-STRPCA to illustrate the effectiveness of generalized-SVD. The results of the single-cell multi-omics data clustering experiments show that the clustering accuracy of GSTRPCA is superior to the other state-of-the-art methods probably because it can reduce redundant features and capture the global subspace and local geometric structure of the data. In summary, the GSTRPCA method can better handle single-cell multi-omics data and detect multiple omics layers in a single cell. This method provides a comprehensive and accurate understanding of cellular information, thereby promoting downstream clustering analysis and enrichment analysis.

The results in this paper are customised for the third-order irregular tensor. However, the single-cell multi-omics data in the real world are partially represented as $d$-order irregular tensors (usually $d\ge 4$). For instance, single-cell multi-omics introduces time and spatial factors, resulting in higher-order irregular tensors. Therefore, in the future, a key focus of our research will be to explore how to extend GSTRPCA to accommodate higher-order irregular tensors or to investigate ways to decompose higher-order irregular tensors into third-order irregular tensors, thereby making our results applicable to these complex data structures.

**Key Points**

- The paper proposed a new model that introduces irregular tensor decomposition and clustering into the field of single-cell multi-omics data.
- The paper developed a new method (GSTRPCA) and compared with the state-of-the-art clustering model. Aiming at the shortcomings of previous models, a weighted threshold model for irregular tensor data decomposition is proposed by integrating low rank and sparse constraints.
- We also conducted theoretical convergence analysis on the algorithm, and compared the iterative error curves between GSTRPCA and the competing algorithms to confirm the superior convergence rate of our method.
- Experimental results have shown that GSTRPCA has excellent predictive and generalization ability.

## Supplementary Material

Supp_clean_bbae649
