# Supplementary material for: GSTRPCA: irregular tensor singular value decomposition for single-cell multi-omics data clustering
Source: Brief Bioinform. 2024 Dec 16;26(1):bbae649. doi: 10.1093/bib/bbae649 (PMC11647523; doi:10.1093/bib/bbae649)
Supplement: Supp_clean_bbae649 [file supp_clean_bbae649.pdf]

Supplementary materials

Generalized Singular Value Decomposition (GSVD) algorithm

For the original data $\mathcal{X} \in \mathbb{R}^{N_i \times M \times K}$, the slice matrix dimension of a tensor $\mathcal{X}$ is $N_i \times M$, each row dimension of the matrix data may be different and dimensions of the columns are the same. When performing singular value decomposition, GSVD algorithms are used to calculate singular values. The difference between GSVD algorithm and traditional SVD algorithm is that, GSVD does not directly perform singular value algorithm on each positive slice matrix $\mathcal{X}(:,:,i)$ of $\mathcal{X}$. Instead, it first preprocesses the positive slice matrix $\mathcal{X}(:,:,i)$, transposes the slice matrix product, and then calculates the arithmetic mean to obtain a common subspace matrix S of the same size. Then, its eigenvector matrix V is calculated for S, then U, Σ are obtained, and hence obtain an approximate estimate of SVD.
The main steps for GSVD are as follows:

1. Define $D_i \in \mathbb{R}^{N_i \times M}$ as follows:

$$
\begin{aligned}
D_1 &= U_1 \Sigma_1 V^T, \\
D_2 &= U_2 \Sigma_2 V^T, \\
&\vdots \\
D_N &= U_N \Sigma_N V^T.
\end{aligned}
$$

2. Define the common subspace matrix S for these matrices.

$$
\begin{aligned}
A_i &= D_i^T D_i, \\
S_{ij} &= \frac{1}{2}(A_i A_j^{-1} + A_j A_i^{-1}), \quad i \neq j
\end{aligned}
$$

$$
\begin{aligned}
S &\equiv \frac{1}{N(N-1)} \sum_{i=1}^{N} \sum_{j>i}^{N} (A_i A_j^{-1} + A_j A_i^{-1}) \\
&= \frac{2}{N(N-1)} \sum_{i=1}^{N} \sum_{j>i}^{N} S_{ij}.
\end{aligned}
$$

3. Obtain the singular value decomposition U, Σ, V from the matrix S constructed above.

$$
\begin{aligned}
SV &= V\Lambda, V = (v_1, v_2, \ldots, v_n), \\
\Lambda &= \mathrm{diag}(\lambda_k).
\end{aligned}
$$

after giving V, obtain U and Σ by calculating matrix B:

$$
\begin{aligned}
VB_i^T &= D_i^T, \\
B_i &\equiv (b_{i,1}, \ldots, b_{i,n}), \quad i = 1, 2, \ldots, N, \\
\sigma_{i,k} &= \|b_{i,k}\|, \\
\Sigma_i &= \mathrm{diag}(\sigma_{i,k}), \\
B_i &= U_i \Sigma_i.
\end{aligned}
$$

Through the above calculations, singular value decomposition can be obtained for N matrices D_i with different dimensions. In the decomposition, all matrices have the same V but different U, Σ.

Irregular tensor nuclear norm(ITNN)

For irregular tensors $\mathcal{X} \in \mathbb{R}^{N_i \times M \times K}$, the nuclear norm, which represents the sum of singular values, can be effectively computed using Generalized Singular Value Decomposition (GSVD). While Singular Value Decomposition (SVD) is typically suitable for square matrices, GSVD accommodates non-square and sparse matrices, making it advantageous for numerical stability in high-dimensional and sparse datasets discussed in this context. Based on GSVD, the nuclear norm of the new irregular tensor (ITNN) in this paper is defined as:

$$
\|\mathcal{X}\|_* = \sum_{k=1}^{K} \|\mathcal{X}(:,:,i)\|_* = \sum_{k=1}^{K} \sum_{j} |\sigma_j(\mathcal{X}(:,:,i))|.
$$

here, $\sigma_j(\mathcal{X}(:,:,i))$ denotes the i-th singular value of $\mathcal{X}(:,:,i)$.

Based on the aforementioned GSVD algorithm and nuclear norm definition, the following properties hold for the irregular tensors discussed in this paper:

(1) Positive definiteness

For any irregular tensor $\mathcal{X}$, we have $\|\mathcal{X}\|_* \geq 0$,

Since the generalized singular values σ obtained from the GSVD are derived from B and are non-negative, their summation is also non-negative. $\|\mathcal{X}\|_* = 0$ if and only if $\mathcal{X} = 0$.

(2) Homogeneity

For any real number a and irregular tensor $\mathcal{X}$, we assume that the irregular tensor $\mathcal{X}$ is composed of k matrices X, Define $X_i \in \mathbb{R}^{N_i \times M}$. For $a\mathcal{X}$, we have

$$
\begin{aligned}
A_i &= aX_i^T \times aX_i, \\
S_{ij} &= \frac{1}{2}(a^2 A_i \times (a^2 A_j)^{-1} + a^2 A_j \times (a^2 A_i)^{-1}) \\
&= \frac{1}{2}(A_i A_j^{-1} + A_j A_i^{-1}), \quad i \neq j
\end{aligned}
$$

When calculating the singular values we get

$$
\begin{aligned}
B_i^T &= aX_i V^{-T}, \\
\sigma_{i,k} &= \|b_{i,k}\|.
\end{aligned}
$$

For $\mathcal{X}$, we have

$$
\begin{aligned}
B_i^T &= X_i V^{-T}, \\
\sigma_{i,k} &= \|b_{i,k}\|.
\end{aligned}
$$

Therefore, we get

$$
\|a\mathcal{X}\|_* = |a| \, \|\mathcal{X}\|_*.
$$

(3) Trigonometric inequality

For any irregular tensor $\mathcal{X}$ and $\mathcal{Y}$, in the section "Convergence analytic decomposition of irregular tensor decompositions" we proved that $\|\mathcal{X} + \mathcal{Y}\|_* \leq \|\mathcal{X}\|_* + \|\mathcal{Y}\|_*$.

By verifying that the generalized singular value decomposition (GSVD)-based nuclear norm satisfies all the defining properties, we can confirm that this nuclear norm is a valid and consistent definition for tensors.

Optimizing $\mathcal{L}$, $\mathcal{E}$, $\mathcal{Y}$ and μ

In the following, we try to optimize $\mathcal{L}$ and $\mathcal{E}$ using the ADMM for the GSTRPCA method. For irregular tensor $\mathcal{X} \in$

$\mathbb{R}^{N_i \times M \times K}$, based on the L_1 norm, the objective function of TRPCA is as follows:

$$\begin{aligned} \min_{\mathcal{L},\mathcal{E}} \quad & \|\mathcal{L}\|_* + \lambda \|\mathcal{E}\|_1 \\ s.t. \quad & \mathcal{X} = \mathcal{L} + \mathcal{E}, \end{aligned} \tag{3.1}$$

To solve the objective function, we encounter a polynomial optimization challenge. Then we introduces $\|\mathcal{E}\|_1$ as the norm for regularization of sparse terms, employing the ADMM algorithm to achieve the model's optimal solution. Augmented Lagrangian multipliers are utilized to reformulate Equation (3.1), the objective function becomes:

$$\begin{aligned} P(\mathcal{L},\mathcal{E},\mathcal{Y},\mu) = & \|\mathcal{L}\|_* + \lambda \|\mathcal{E}\|_1 + < \mathcal{Y}, \mathcal{L} + \mathcal{E} - \mathcal{X} > \\ & + \frac{\mu}{2} \|\mathcal{L} + \mathcal{E} - \mathcal{X}\|_F^2 , \end{aligned} \tag{3.2}$$

where $\mathcal{Y}$ is the dual variable and μ is the introduced equilibrium parameter.

Under the ADMM framework, the formula for updating variables $\mathcal{L}^{k+1}$ and $\mathcal{E}^{k+1}$ can be expressed in the following forms:

$$\begin{aligned} \mathcal{L}^{k+1} & = \arg\min_{\mathcal{L}} P(\mathcal{L},\mathcal{E}^k,\mathcal{Y}^k,\mu^k) \\ & = \arg\min_{\mathcal{L}} \|\mathcal{L}\|_* + \frac{\mu^k}{2} \left\| \mathcal{L} + \mathcal{E}^k - \mathcal{X} + \frac{\mathcal{Y}^k}{\mu^k} \right\|_F^2 , \end{aligned} \tag{3.3}$$

$$\begin{aligned} \mathcal{E}^{k+1} & = \arg\min_{\mathcal{E}} P(\mathcal{L}^{k+1},\mathcal{E},\mathcal{Y}^k,\mu^k) \\ & = \arg\min_{\mathcal{E}} \lambda \|\mathcal{E}\|_1 + \frac{\mu^k}{2} \left\| \mathcal{L}^{k+1} + \mathcal{E} - \mathcal{X} + \frac{\mathcal{Y}^k}{\mu^k} \right\|_F^2 . \end{aligned} \tag{3.4}$$

Then update irregular low-rank tensor $\mathcal{L}$:

1. Apply the Generalized Singular Value Thresholding (GSVT) algorithm to the frontal slice $\mathcal{X}(:,:,i)$ of each irregular tensor data $\mathcal{X}$

$$\left\{U^{(i)}, S^{(i)}, V^{(i)}\right\}_{i=1}^{K} = GSVD(\mathcal{X}(:,:,i)). \tag{3.5}$$

2. Construct threshold factors related to matrix S

$$C = \max[S^{(i)} - \frac{1}{(S^{(i)} - \xi)^{1-p}}, 0]. \tag{3.6}$$

3. Construct an irregular low-rank tensor $\mathcal{L}$

$$\mathcal{L}(:,:,i) = U^{(i)} \times C^{(i)} \times (V^{(i)})^T. \tag{3.7}$$

Following the above process, the updating rule for $\mathcal{L}$ can be obtained as follows:

$$\mathcal{L}^{k+1} = D_{\mathcal{L}} \left(\mathcal{X} - \mathcal{E}^k - \frac{\mathcal{Y}^k}{\mu^k} \right). \tag{3.8}$$

When seeking the optimal solution for $\mathcal{E}^{k+1}$, we fix the irregular low-rank tensor $\mathcal{L}^{k+1}$ and Lagrange multiplier $\mathcal{Y}^k$, which are not updated temporarily. Solving this optimization problem requires the use of threshold processing operations.

$$\mathcal{E}^{k+1} = prox_{\mu} \left(\mathcal{X} - \mathcal{L}^{k+1} - \frac{\mathcal{Y}^k}{\mu^k} \right), \tag{3.9}$$

$$prox_{\mu}(x) = \max(x - \mu, 0) + \min(x + \mu, 0).$$

Afterwards, update the dual variable $\mathcal{Y}^{k+1}$:

$$\mathcal{Y}^{k+1} = \mathcal{Y}^k + \mu^k \times (\mathcal{L}^{k+1} + \mathcal{E}^{k+1} - \mathcal{X}). \tag{3.10}$$

After completing one iteration update of the above variables, establish a new balancing parameter μ^{k+1} for the subsequent iteration update and adjust it using a fixed step size.

$$\mu^{k+1} = \min(\rho\mu^k, \mu^{max}). \tag{3.11}$$

The framework of the algorithm is shown as Algorithm 1.

Algorithm 1 GSTRPCA Algorithm

Input: Given $\mathcal{X} \in \mathbb{R}^{N_i \times M \times K}$, $\mu = 1e-5$, ξ, $\mu^{max} = 1e+5$ and set $k = 1$;

1: **While** certain stopping criterion is not reached, do

2: Update primary variable

$$\mathcal{L}^{k+1} = D_{\mathcal{L}} \left(\mathcal{L} - \mathcal{E}^k - \frac{\mathcal{Y}^k}{\mu^k} \right).$$

$$\mathcal{E}^{k+1} = prox_{\mu} \left(\mathcal{X} - \mathcal{L}^{k+1} - \frac{\mathcal{Y}^k}{\mu^k} \right).$$

3: Compute the lagrangian multipliers

$$\mathcal{Y}^{k+1} = \mathcal{Y}^k + \mu^k(\mathcal{L}^{k+1} + \mathcal{E}^{k+1} - \mathcal{X}).$$

4: Update step size

$$\mu^{k+1} = \min(\rho\mu^k, \mu^{max}).$$

5: Set $k := k + 1$.

Output: $\mathcal{L}^{\mathbf{k+1}}$,$\mathcal{E}^{\mathbf{k+1}}$.

Parameter analysis

In the study of this paper, we use the threshold parameter p in Eq.(3.6) to construct the singular value matrix in irregular low-rank tensor decomposition. This approach allows for a significant penalization of very small singular values, effectively diminishing the impact of noise on data analysis. In our experiments, the choice of the singular value truncation parameter p affects the results of our method, and we go through the results of the subsequent downstream cluster analyses to determine the final choice of parameter p. In the GSTRPCA model, we control the range of values within [0,1) by grid search. Experimental parameter selection across various datasets yields results as depicted in Figure 1. Parameter analysis for five single-cell multi-omics datasets enables the determination of optimal algorithm settings. In the solution of the irregular low-rank tensor presented in Eq.(3.6), the parameter p is used to control the sensitivity to the data characteristics. For instance, the real dataset $10X\_inhouse$, the

parameter threshold p is selected to be 0.25, this is because the two types of data size is too different and data sparse, when the value of p is taken to a smaller value, it will maximise the suppression of the noise during the data processing process, which is usually used in data If the value of p is 0, then the behaviour of the shrinkage function will be too strong leading to the destruction of the data structure, so the inappropriate value of p will greatly affect the performance of the subsequent prediction. We determined the optimal parameter selection for different datasets through clustering experiments, details are shown in Table 1.

During the iterative process, the augmented lagrangian function is used to update alternately. In each updating step, we calculate the errors separately for the low-rank component and the sparse component. The algorithm achieves convergence when errors are less than a predefined minimum error. Figure 2 compares the convergence curves of GSTRPCA and the competing methods on five datasets. We can see that the errors of GSTRPCA are much less than the ones of other methods on these five datasets. In particular, GSTRPCA converges in the third iteration on the real datasets, indicating that GSTRPCA is much more precise and faster than the typical methods. Therefore, we can conclude that GSTRPCA accelerates the speed of convergence.

Time analysis

Time analysis is the process for evaluating the efficiency of the algorithms. Table 2 compares the running time of GSTRPCA and the competing methods on different datasets and we can see that the running time of the GSTRPCA is much less than the ones for other methods.

Feature selection

On the Specter dataset, we utilize Principal Component Analysis (PCA) [1] to select the top 50 significant genes from the irregular sparse tensor $\mathcal{E}$ obtained through GSTRPCA decomposition. The main steps for PCA are as follows:

1. Normalize the raw data.
2. Compute the covariance matrix of the standardized data.
3. Perform eigenvalue decomposition on the covariance matrix to obtain eigenvalues (representing variance) and eigenvectors (representing principal components).
4. Select principal components based on the magnitude of their eigenvalues, where larger eigenvalues correspond to capturing more data variance.
5. Compute scores for each sample on the selected principal components.

Table 3 summarizes detailed information on the top 50 co-expressed genes identified through PCA in the Specter dataset. Figure 3 presents a heatmap depicting the expression profiles of the top 50 co-expressed genes. In this heatmap, the color gradient transitions from left to right, indicating relative gene expression levels, with the left side representing high expression and the right side representing low expression. The rows correspond to different genes.

Convergence analysis for irregular tensor decomposition

For the iterative updating of irregular tensors $\mathcal{L}$ and $\mathcal{E}$, the updating rule at each step determines the global convergence of the algorithm. In general ADMM alternating iterations, proximal alternating linearization minimization methods establish the global convergence of algorithms through the concept of auxiliary functions in their update rules [2]. Sparse coding algorithms demonstrate their global convergence through proofs of error boundedness, continuity, and monotonicity [3]. In semi-algebraic problems, the regularized Gauss-Seidel method is a common iterative solving technique used for solving systems of linear equations [4]. Based on the $Kurdyka - Łojasiewicz$ property, a convergence analysis framework is derived [5].

The objective function for irregular tensor decomposition is as follows:

$$\min_{\mathcal{L},\mathcal{E}} \ \|\mathcal{L}\|_* + \lambda \|\mathcal{E}\|_1$$

$$s.t. \ \ \mathcal{X} = \mathcal{L} + \mathcal{E},$$

where, $\mathcal{X} \in \mathbb{R}^{N_i \times M \times K}$, $\mathcal{L} \in \mathbb{R}^{N_i \times M \times K}$, $\mathcal{E} \in \mathbb{R}^{N_i \times M \times K}$. Now, we introduce the KŁ function used to prove the convergence of the Algorithm 1.

Definition 1 ($Kurdyka$-$Łojasiewicz$ $property$ [5]): Let $f : R^d \longrightarrow (-\infty, +\infty]$ be proper and lower semi-continuous. We have the KŁ property at $\bar{x} \in dom(\partial f) := \left\{x \in R^d : \partial f \neq \emptyset\right\}$ if there exist $\eta \in (0, +\infty]$, a neighborhood U of $\bar{x}$, and a continuous concave function $\varphi : [0, \eta) \longrightarrow [0, +\infty)$ such that:

(1)$\varphi(0) = 0$;
(2)φ is C^1 on $(0, \eta)$ and continuous at 0;
(3)$\varphi'(s) > 0$, $s \in (0, \eta)$;
Such that for all

$$u \in U \cap [f(\bar{x}) < f(x) < f(\bar{x}) + \eta],$$

the following inequality holds:

$$\varphi'(f(x) - f(\bar{x}))dist(0, \partial f(x)) \geq 1.$$

If f satisfies the KŁ property at each point of dom (∂f), then f is called a KŁ function.

The principal instrument for the proof is the following theorem.

Theorem 1: Assuming the existence of sequence $\left\{\mathcal{L}^k, \mathcal{E}^k\right\}$ is KŁ function. If the sequence $\left\{\mathcal{L}^k, \mathcal{E}^k\right\}$ from Algorithm 1 satisfies the following conditions, then the sequence will globally converge to a critical point of equation (3.2).

(1) The function $P(\mathcal{L}, \mathcal{E}, \mathcal{Y}, \mu)$ satisfies the KŁ property at each point.

(2) The sequence $\left\{\mathcal{L}^k, \mathcal{E}^k\right\}$ satisfies the sufficient decrease property.

(3) The sequence $\left\{\mathcal{L}^k, \mathcal{E}^k\right\}$ satisfies the Relative error property.

(4) The sequence $\left\{\mathcal{L}^k, \mathcal{E}^k\right\}$ satisfies the continuity property.

Before verifying these conditions, we provide following lemmas.

In Lemma 1, we establish that the $\|\cdot\|_1$ defined based on irregular tensors satisfies convexity properties.

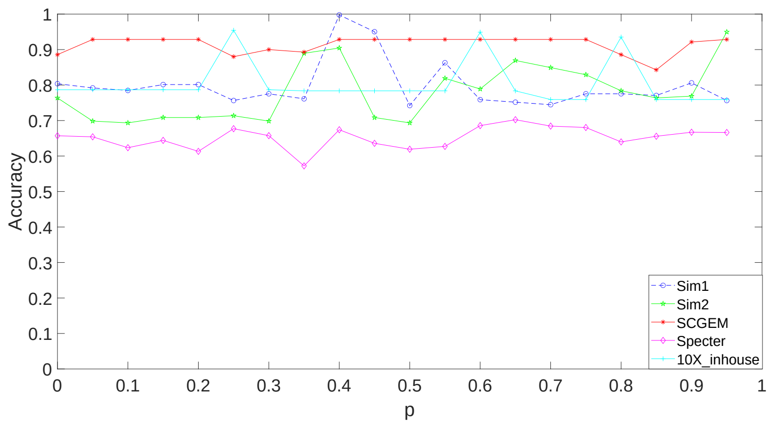

Fig. 1. Sensitivity analysis of GSTRPCA parameter p in five different dataset.

Table 1. The determination for p on different datasets.

Dataset	Parameter p	ACC	ARI	AMI	NMI
Sim1	0.45	99.76	99.28	99.06	99.08
Sim2	0.95	94.98	88.67	91.35	91.57
SCGEM	0.45	92.86	82.76	83.86	84.47
Specter	0.65	70.24	56.83	68.37	68.83
10X_inhouse	0.25	95.44	92.28	92.25	92.24

Table 2. Run time(s) analysis of GSTRPCA and the competing methods on five datasets.

	LRTV	LLRGTV	t-TRPCA	LRTD	TT-TRPCA	GSTRPCA
Sim1	7.21	83.47	34.72	97.53	59.17	**5.75**
Sim2	6.42	69.65	21.34	127.65	29.70	**0.92**
SCGEM	0.11	2.11	0.18	2.38	0.16	**0.05**
Specter	23.89	137.89	176.81	160.28	155.87	**21.67**
10X_inhouse	101.26	799.17	442.07	828.95	836.59	**27.84**

Table 3. The top 50 genes with the highest scores on the Specter dataset and their scores.

Rank	Gene	Score	Rank	Gene	Score
1	hg19_MED8	41.66	2	hg19_RPA2	38.94
3	hg19_ZFP69	35.32	4	hg19_KDM1A	35.18
5	hg19_LUZP1	34.11	6	hg19_TMEM69	29.61
7	hg19_CAPZB	29.59	8	hg19_NMNAT1	27.52
9	hg19_OTUD3	27.01	10	hg19_C1orf167	26.89
11	hg19_EXTL1	26.62	12	hg19_LINC01715	25.16
13	hg19_KIAA1522	23.29	14	hg19_TMEM50A	23.07
15	hg19_LINC01646	22.91	16	hg19_SLC25A34	22.78
17	hg19_LINC01648	22.74	18	hg19_SLC6A9	22.19
19	hg19_NUDC	21.44	20	hg19_PADI2	20.94
21	hg19_CATSPER4	20.17	22	hg19_CMPK1	19.52
23	hg19_RNF207	19.37	24	hg19_BTBD19	19.27
25	hg19_DRAXIN	18.98	26	hg19_KIAA2013	18.93
27	hg19_FAM183A	18.81	28	hg19_PADI4	18.58
29	hg19_GPR3	18.26	30	hg19_PHF13	18.13
31	hg19_ATAD3B	17.36	32	hg19_TPRG1L	16.92
33	hg19_KCNQ4	15.71	34	hg19_LINC01141	15.59
35	hg19_PADI1	15.53	36	hg19_PLA2G2D	15.33
37	hg19_GPN2	15.19	38	hg19_ANKRD65	15.12
39	hg19_CROCC	14.83	40	hg19_RAB42	14.72
41	hg19_FAM138A	14.66	42	hg19_KHDRBS1	14.64
43	hg19_NCDN	14.40	44	hg19_UTP11	14.39
45	hg19_FAM229A	14.38	46	hg19_TAS1R2	13.81
47	hg19_ICMT	13.67	48	hg19_TNFRSF14-AS1	13.60
49	hg19_ZBTB48	13.51	50	hg19_MINOS1	13.46

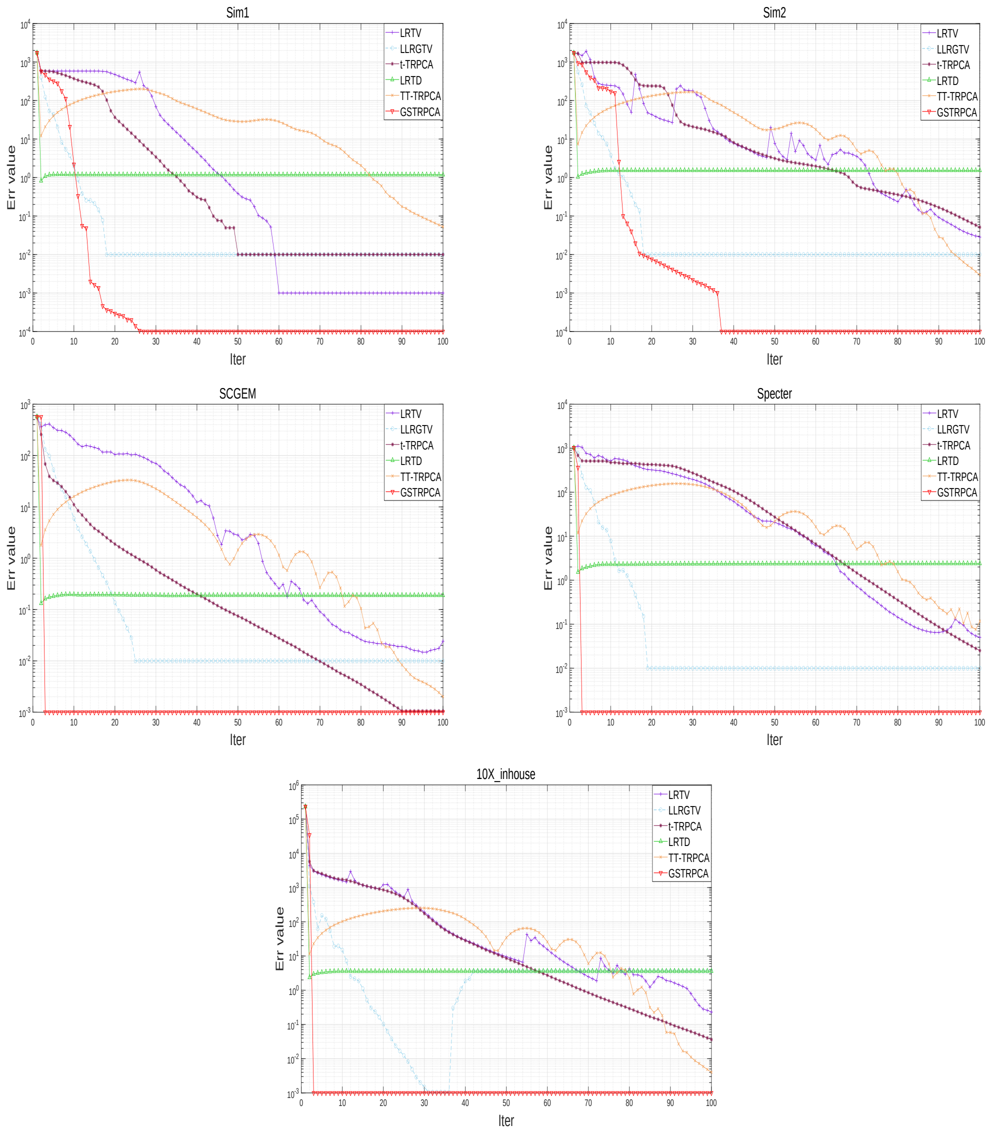

Fig. 2. The convergence speed of LRTV, LLRGVT, t-TRPCA, LRTD, TT-TRPCA and GSTRPCA when the number of iterations increases from 1 to 100 on five datasets.

Lemma 1 : The $\|\cdot\|_1$ defined in this paper is a convex function.

Proof The $\|\cdot\|_1$ is defined as:

$$\|\mathcal{X}\|_1 = \sum_{i_1=1}^{N_i} \sum_{i_2=1}^{M} \sum_{i_3=1}^{K} |x_{i_1 i_2 i_3}| \,.$$

Where, $x_{i_1 i_2 i_3}$ represents an element of the irregular tensor X at a specific index, with N_i, M and K denoting the sizes of the tensor along each respective dimension.

(1) Positive definiteness

For any irregular tensor $\mathcal{X}$, the sum of the absolute values of each element is non-negative, therefore, $\|\mathcal{X}\|_1 \geq 0$.

(2) Homogeneity

For any real number k and irregular tensor $\mathcal{X}$, we have

$$\|k\mathcal{X}\|_1 = \sum_{i_1=1}^{N_i} \sum_{i_2=1}^{M} \sum_{i_3=1}^{K} |kx_{i_1 i_2 i_3}| \,.$$

$$= |k| \sum_{i_1=1}^{N_i} \sum_{i_2=1}^{M} \sum_{i_3=1}^{K} |x_{i_1 i_2 i_3}| \,. = |k| \, \|\mathcal{X}\|_1$$

(3) Trigonometric inequality

For any two irregular tensors $\mathcal{X}$ and $\mathcal{Y}$, we have

$$\|\mathcal{X} + \mathcal{Y}\|_1 = \sum_{i_1=1}^{N_i} \sum_{i_2=1}^{M} \sum_{i_3=1}^{K} |x_{i_1 i_2 i_3} + y_{i_1 i_2 i_3}| \,.$$

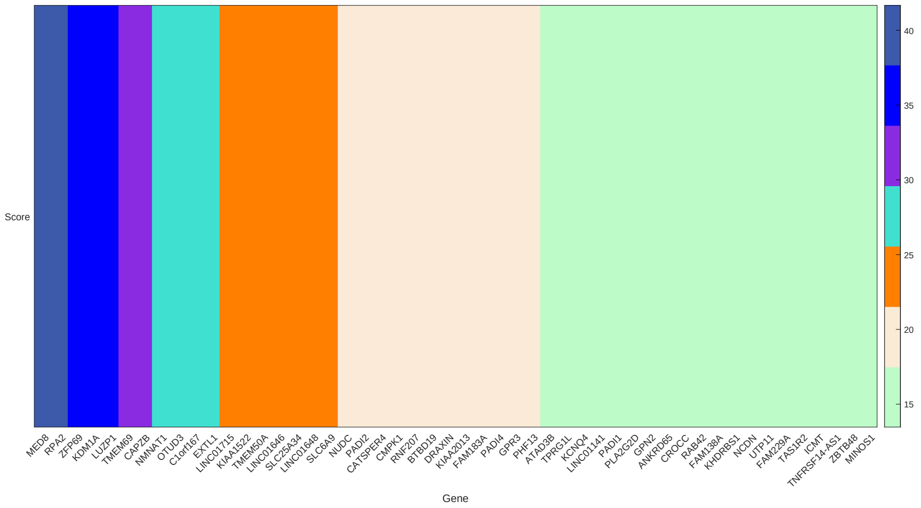

Fig. 3. Statistical Analysis Results of the Heatmap for top 50 genes.

Applying absolute value trigonometric inequalities, then

$$\begin{aligned}\|\mathcal{X}+\mathcal{Y}\|_1 &\le \sum_{i_1=1}^{N_i}\sum_{i_2=1}^{M}\sum_{i_3=1}^{K}(|x_{i_1i_2i_3}|+|y_{i_1i_2i_3}|)\\ &\le \sum_{i_1=1}^{N_i}\sum_{i_2=1}^{M}\sum_{i_3=1}^{K}|x_{i_1i_2i_3}|+\sum_{i_1=1}^{N_i}\sum_{i_2=1}^{M}\sum_{i_3=1}^{K}|y_{i_1i_2i_3}|\\ &= \|\mathcal{X}\|_1+\|\mathcal{Y}\|_1 .\end{aligned}$$

We have proved that the $\|\cdot\|_1$ satisfies three properties of the norm. Therefore, the $\|\cdot\|_1$ is a norm. Obviously, the $\|\cdot\|_1$ is a convex function. □

We use GSVD to establish $\|L\|_*$. In Lemma 2 we prove the convexity property of $\|L\|_*$. The $\|\cdot\|_*$ is defined as follows: utilizing the common subspace S, which is derived from solving K linear systems based on the eigenvalues of S and the irregular tensor data X to calculate matrix B. The columns of B are normalized to obtain the sum of singular values of the irregular tensor.

Lemma 2 : The $\|\cdot\|_*$ defined for generalized singular value decomposition in this paper is a convex function.

Proof Based on the irregular tensor, we take $\mathcal{X}\in\mathbb{R}^{(N_1,N_2,\ldots,N_K)\times M\times K}$, $\mathcal{Y}\in\mathbb{R}^{(N_1,N_2,\ldots,N_K)\times M\times K}$, $\mathcal{Z}\in\mathbb{R}^{(N_1,N_2,\ldots,N_K)\times M\times K}$, and $\mathcal{Z}=\lambda\mathcal{X}+(1-\lambda)\mathcal{Y}, \lambda\in(0,1)$.

According to the definition of generalized singular value decomposition, we have

$$\begin{aligned}A_{\mathcal{X}} &= \mathcal{X}(:,:,i)^T\times\mathcal{X}(:,:,i),\\ A_{\mathcal{Y}} &= \mathcal{Y}(:,:,i)^T\times\mathcal{Y}(:,:,i),\\ A_{\mathcal{Z}} &= \mathcal{Z}(:,:,i)^T\times\mathcal{Z}(:,:,i)\\ &= \lambda^2\mathcal{X}(:,:,i)^T\times\mathcal{X}(:,:,i)+((1-\lambda)\mathcal{Y}(:,:,i))^T\times\lambda\mathcal{X}(:,:,i)\\ &+(\lambda\mathcal{X}(:,:,i))^T\times(1-\lambda)\mathcal{Y}(:,:,i)\\ &+(1-\lambda)^2\mathcal{Y}(:,:,i)^T\times\mathcal{Y}(:,:,i).\end{aligned}$$

Calculate S and normalize the columns of S, then we have

$$\begin{aligned}S_{\mathcal{X}} &= \frac{1}{K(K-1)}\sum_{i=1}^{K}\sum_{j>i}^{K}(A_{\mathcal{X}}(:,:,i)A_{\mathcal{X}}(:,:,j)^{-1}+\\ &A_{\mathcal{X}}(:,:,j)A_{\mathcal{X}}(:,:,i)^{-1}),\\ S_{\mathcal{Y}} &= \frac{1}{K(K-1)}\sum_{i=1}^{K}\sum_{j>i}^{K}(A_{\mathcal{Y}}(:,:,i)A_{\mathcal{Y}}(:,:,j)^{-1}+\\ &A_{\mathcal{Y}}(:,:,j)A_{\mathcal{Y}}(:,:,i)^{-1}),\\ S_{\mathcal{Z}} &= \frac{1}{K(K-1)}\sum_{i=1}^{K}\sum_{j>i}^{K}(A_{\mathcal{Z}}(:,:,i)A_{\mathcal{Z}}(:,:,j)^{-1}+\\ &A_{\mathcal{Z}}(:,:,j)A_{\mathcal{Z}}(:,:,i)^{-1}).\end{aligned}$$

Compute the right singular vectors V and eigenvalues Λ of $\mathcal{X}$, $\mathcal{Y}$, and $\mathcal{Z}$, normalize V, where the eigenvalues Λ and eigenvectors V are real numbers[6].

$$\begin{aligned}S_{\mathcal{X}}V_{\mathcal{X}} &= V_{\mathcal{X}}\Lambda_{\mathcal{X}},\\ S_{\mathcal{Y}}V_{\mathcal{Y}} &= V_{\mathcal{Y}}\Lambda_{\mathcal{Y}},\\ S_{\mathcal{Z}}V_{\mathcal{Z}} &= V_{\mathcal{Z}}\Lambda_{\mathcal{Z}}.\end{aligned}$$

Where $\|V_{\mathcal{X}}\|=1$, $\|V_{\mathcal{Y}}\|=1$, $\|V_{\mathcal{Z}}\|=1$. And the eigenvalues satisfy

$$\begin{aligned}\sum_{i=1}^{M}\Lambda_{\mathcal{Z}}(i,i) &= \sum_{i=1}^{M}\Lambda_{\lambda\mathcal{X}+(1-\lambda)\mathcal{Y}}(i,i)\\ &\le \lambda\sum_{i=1}^{M}\Lambda_{\mathcal{X}}(i,i)+(1-\lambda)\sum_{i=1}^{M}\Lambda_{\mathcal{Y}}(i,i).\end{aligned}$$

After obtaining V, we compute $\mathcal{B}$ by solving K linear systems:

$$\begin{aligned}V_{\mathcal{X}}\mathcal{B}_{\mathcal{X}}^T &= \mathcal{X}(:,:,i)^T,\\ V_{\mathcal{Y}}\mathcal{B}_{\mathcal{Y}}^T &= \mathcal{Y}(:,:,i)^T,\\ V_{\mathcal{Z}}\mathcal{B}_{\mathcal{Z}}^T &= \mathcal{Z}(:,:,i)^T.\end{aligned}$$

Normalize the columns of $\mathcal{B}$ to obtain matrix σ, and then derive the singular value matrix Σ

$$\begin{aligned}
\sigma_{\mathcal{X}_i} &= \|\mathcal{B}_{\mathcal{X}}\|, \\
\sigma_{\mathcal{Y}_i} &= \|\mathcal{B}_{\mathcal{Y}}\|, \\
\sigma_{\mathcal{Z}_i} &= \|\mathcal{B}_{\mathcal{Z}}\|, \\
\Sigma_{\mathcal{X}} &= diag(\sigma_{\mathcal{X}_i}), \\
\Sigma_{\mathcal{Y}} &= diag(\sigma_{\mathcal{Y}_i}), \\
\Sigma_{\mathcal{Z}} &= diag(\sigma_{\mathcal{Z}_i}).
\end{aligned}$$

By using the eigenvalue relationship of $\mathcal{X}, \mathcal{Y}$, and $\mathcal{Z}$, the corresponding singular values can be obtained as follows:

$$\begin{aligned}
\sum_{i=1}^{M} \Sigma_{\mathcal{Z}}(i,i) &= \sum_{i=1}^{M} \Sigma_{\lambda\mathcal{X}+(1-\lambda)\mathcal{Y}}(i,i) \\
&\leq \lambda \sum_{i=1}^{M} \Sigma_{\mathcal{X}}(i,i) + (1-\lambda) \sum_{i=1}^{M} \Sigma_{\mathcal{Y}}(i,i), \lambda \in (0,1).
\end{aligned}$$

Based on the above inequality, we can conclude that the $\|\cdot\|_*$ is a convex function. □

Now, we prove the convergence analysis of the proposed algorithm as follows.

Lemma 3 ($K Ł$ Lemma): function $P(\mathcal{L}, \mathcal{E}, y, \mu)$ satisfies the $K Ł$ property at each point.

Proof Based on Lemma 1 and Lemma 2, we have demonstrated that the objective function is convex. A convex function can be regarded as a semi-algebraic function, inherently satisfying the so-called $K Ł$ property [5]. □

Next, we show $P(\mathcal{L}, \mathcal{E}, \mathcal{Y}, \mu)$ satisfies three main lemmas.

Lemma 4 (Sufficient decrease property): there exists positive constant ρ_1, ρ_2, such that

$$\begin{aligned}
&P(\mathcal{L}^{k+1}, \mathcal{E}^{k+1}, \mathcal{Y}^k, \mu^k) - P(\mathcal{L}^{k+1}, \mathcal{E}^k, \mathcal{Y}^k, \mu^k) \\
&\leq -\rho_1 \left\|\mathcal{E}^{k+1} - \mathcal{E}^k\right\|_F^2, \\
&P(\mathcal{L}^{k+1}, \mathcal{E}^k, \mathcal{Y}^k, \mu^k) - P(\mathcal{L}^k, \mathcal{E}^k, \mathcal{Y}^k, \mu^k) \\
&\leq -\rho_2 \left\|\mathcal{L}^{k+1} - \mathcal{L}^k\right\|_F^2.
\end{aligned}$$

Proof According to the definition of $P(\mathcal{L}, \mathcal{E}, y, \mu)$ in Algorithm 1, we have

$$\begin{aligned}
P(\mathcal{L}, \mathcal{E}, \mathcal{Y}, \mu) = \|\mathcal{L}\|_* + \lambda \|\mathcal{E}\|_1 + \frac{\mu}{2} \left\|\mathcal{L} + \mathcal{E} - \mathcal{X} + \frac{\mathcal{Y}}{\mu}\right\|_F^2 \\
- \frac{\mu}{2} \left\|\frac{\mathcal{Y}}{\mu}\right\|_F^2.
\end{aligned}$$

Therefore, we have

$$\begin{aligned}
&P(\mathcal{L}^{k+1}, \mathcal{E}^{k+1}, \mathcal{Y}^k) - P(\mathcal{L}^{k+1}, \mathcal{E}^k, \mathcal{Y}^k) \\
&= \lambda \left\|\mathcal{E}^{k+1}\right\|_1 + \frac{\mu^k}{2} \left\|\mathcal{L}^{k+1} + \mathcal{E}^{k+1} - \mathcal{X} + \frac{\mathcal{Y}^k}{\mu^k}\right\|_F^2 \\
&- \lambda \left\|\mathcal{E}^k\right\|_1 - \frac{\mu^k}{2} \left\|\mathcal{L}^{k+1} + \mathcal{E}^k - \mathcal{X} + \frac{\mathcal{Y}^k}{\mu^k}\right\|_F^2 \\
&= \mu^k < \mathcal{L}^{k+1} - \mathcal{X} + \frac{\mathcal{Y}^k}{\mu^k}, \mathcal{E}^{k+1} - \mathcal{E}^k > + \\
&\lambda(\left\|\mathcal{E}^{k+1}\right\|_1 - \left\|\mathcal{E}^k\right\|_1) + \frac{\mu^k}{2}(\left\|\mathcal{E}^{k+1}\right\|_F^2 - \left\|\mathcal{E}^k\right\|_F^2) \\
&= \lambda(\left\|\mathcal{E}^{k+1}\right\|_1 - \left\|\mathcal{E}^k\right\|_1) + \mu^k < \frac{\mathcal{Y}^k}{\mu^k} - \mathcal{E}^{k+1}, \mathcal{E}^{k+1} - \mathcal{E}^k > \\
&+ \frac{\mu^k}{2}(\left\|\mathcal{E}^{k+1}\right\|_F^2 - \left\|\mathcal{E}^k\right\|_F^2) \\
&\leq -\mu^k < \mathcal{E}^{k+1} - \mathcal{E}^k, (\mathcal{E}^{k+1} - \mathcal{E}^k) + \mathcal{E}^k > \\
&+ \frac{\mu^k}{2}(\left\|\mathcal{E}^{k+1}\right\|_F^2 - \left\|\mathcal{E}^k\right\|_F^2) + \alpha_1(\left\|\mathcal{E}^{k+1}\right\|_2 - \left\|\mathcal{E}^k\right\|_2) \\
&\leq -\mu^k \left\|\mathcal{E}^{k+1} - \mathcal{E}^k\right\|_F^2 + \mu^k < \mathcal{E}^{k+1} - \mathcal{E}^k, -\mathcal{E}^k > \\
&+ \frac{\mu^k}{2} < \mathcal{E}^{k+1} - \mathcal{E}^k, \mathcal{E}^{k+1} + \mathcal{E}^k > + \alpha_1 \left\|\mathcal{E}^{k+1} - \mathcal{E}^k\right\|_2 \\
&\leq -\mu^k \left\|\mathcal{E}^{k+1} - \mathcal{E}^k\right\|_F^2 + \frac{\mu^k}{2} \left\|\mathcal{E}^{k+1} - \mathcal{E}^k\right\|_F^2 + \alpha_1 \left\|\mathcal{E}^{k+1} - \mathcal{E}^k\right\|_F^2 \\
&= -\frac{\mu^k}{2} \left\|\mathcal{E}^{k+1} - \mathcal{E}^k\right\|_F^2 + \alpha_1 \left\|\mathcal{E}^{k+1} - \mathcal{E}^k\right\|_F^2.
\end{aligned}$$

The above three inequalities are applied to absolute value inequality, equivalence of norm, relationship between $\|\cdot\|_2$ and $\|\cdot\|_F^2$. For the norm $\|\cdot\|_\alpha$ and $\|\cdot\|_\beta$ on any two finite dimensional linear spaces V, there exist constant C_1 and C_2, for $\gamma \in V$, such that

$$\begin{aligned}
\|\gamma\|_\alpha \leq C_1 \|\gamma\|_\beta, \\
\|\gamma\|_\beta \leq C_2 \|\gamma\|_\alpha.
\end{aligned}$$

Besides,

$$\begin{aligned}
\|A\| - \|B\| \leq \|A - B\|, \\
\left\|\mathcal{E}^{k+1} - \mathcal{E}^k\right\|_2 \leq \left\|\mathcal{E}^{k+1} - \mathcal{E}^k\right\|_F^2.
\end{aligned}$$

When $\frac{\mu^k}{2} > \alpha$, $\rho_1 = \frac{\mu^k}{2} - \alpha > 0$, we have

$$\begin{aligned}
&P(\mathcal{L}^{k+1}, \mathcal{E}^{k+1}, \mathcal{Y}^k, \mu^k) - P(\mathcal{L}^{k+1}, \mathcal{E}^k, \mathcal{Y}^k, \mu^k) \\
&\leq -\rho_1 \left\|\mathcal{E}^{k+1} - \mathcal{E}^k\right\|_F^2.
\end{aligned}$$

Similarly,

$$\begin{aligned}
&P(\mathcal{L}^{k+1}, \mathcal{E}^k, \mathcal{Y}^k) - P(\mathcal{L}^k, \mathcal{E}^k, \mathcal{Y}^k) \\
&= \left\|\mathcal{L}^{k+1}\right\|_* - \left\|\mathcal{L}^k\right\|_* + \frac{\mu^k}{2} \left\|\mathcal{L}^{k+1} + \mathcal{E}^k - \mathcal{X} + \frac{\mathcal{Y}^k}{\mu^k}\right\|_F^2 \\
&- \frac{\mu^k}{2} \left\|\mathcal{L}^k + \mathcal{E}^k - \mathcal{X} + \frac{\mathcal{Y}^k}{\mu^k}\right\|_F^2 \\
&= \left\|\mathcal{L}^{k+1}\right\|_* - \left\|\mathcal{L}^k\right\|_* + \frac{\mu^k}{2}(\left\|\mathcal{L}^{k+1}\right\|_F^2 - \left\|\mathcal{L}^k\right\|_F^2) \\
&+ \mu^k < \mathcal{L}^{k+1} - \mathcal{L}^k, \mathcal{E}^k - \mathcal{X} + \frac{\mathcal{Y}^k}{\mu^k} > \\
&= \left\|\mathcal{L}^{k+1}\right\|_* - \left\|\mathcal{L}^k\right\|_* + \frac{\mu^k}{2}(\left\|\mathcal{L}^{k+1}\right\|_F^2 - \left\|\mathcal{L}^k\right\|_F^2) \\
&+ \mu^k < \mathcal{L}^{k+1} - \mathcal{L}^k, \frac{\mathcal{Y}^{k+1}}{\mu^k} - \mathcal{L}^{k+1} + \mathcal{E}^k - \mathcal{E}^{k+1} > \\
&\leq \beta_1 \left\|\mathcal{L}^{k+1} - \mathcal{L}^k\right\|_2 + \frac{\mu^k}{2}(\left\|\mathcal{L}^{k+1}\right\|_F^2 - \left\|\mathcal{L}^k\right\|_F^2) \\
&+ \mu^k < \mathcal{L}^{k+1} - \mathcal{L}^k, -\mathcal{L}^{k+1} > + \beta_2 \left\|\mathcal{L}^{k+1} - \mathcal{L}^k\right\|_2
\end{aligned}$$

$$= (\beta_1+\beta_2)\left\|\mathcal{L}^{k+1}-\mathcal{L}^k\right\|_2 + \frac{\mu^k}{2} < \mathcal{L}^{k+1}-\mathcal{L}^k, \mathcal{L}^{k+1}+\mathcal{L}^k >$$
$$- \mu^k < \mathcal{L}^{k+1}-\mathcal{L}^k, \mathcal{L}^{k+1}-\mathcal{L}^k+\mathcal{L}^k >$$
$$\le (\beta_1+\beta_2)\left\|\mathcal{L}^{k+1}-\mathcal{L}^k\right\|_2 + \frac{\mu^k}{2}\left\|\mathcal{L}^{k+1}-\mathcal{L}^k\right\|_F^2$$
$$- \mu^k \left\|\mathcal{L}^{k+1}-\mathcal{L}^k\right\|_F^2$$
$$= (\beta_1+\beta_2)\left\|\mathcal{L}^{k+1}-\mathcal{L}^k\right\|_F^2 - \frac{\mu^k}{2}\left\|\mathcal{L}^{k+1}-\mathcal{L}^k\right\|_F^2.$$

If $\frac{\mu^k}{2} > \beta_1+\beta_2$, when $\rho_2 = \frac{\mu^k}{2} - \beta_1 - \beta_2 > 0$, we have

$$P(\mathcal{L}^{k+1},\mathcal{E}^k,\mathcal{Y}^k,\mu^k) - P(\mathcal{L}^k,\mathcal{E}^k,\mathcal{Y}^k,\mu^k)$$
$$\le -\rho_2\left\|\mathcal{L}^{k+1}-\mathcal{L}^k\right\|_F^2.$$

The proof of the sufficient decrease lemma has been completed. □

Next, we show the sequence generated by the algorithm 1 satisfies the relative error property.

Lemma 5 (Relative error property): find other positive constants ρ_3 and ρ_4, such that

$$\left\|\omega_1^{k+1}\right\|_F \le \rho_3\left\|\mathcal{L}^{k+1}-\mathcal{L}^k\right\|_F, \quad \omega_1^{k+1} \in \partial P(\mathcal{L}^{k+1}),$$
$$\left\|\omega_2^{k+1}\right\|_F \le \rho_4\left\|\mathcal{E}^{k+1}-\mathcal{E}^k\right\|_F, \quad \omega_2^{k+1} \in \partial P(\mathcal{E}^{k+1}).$$

Proof For each subproblem $P_{\mathcal{L}}(\mathcal{L}^{k+1})$ and $P_{\mathcal{E}}(\mathcal{E}^{k+1})$, we have

$$\begin{cases} 0 = \dfrac{\partial(\|\mathcal{L}\|_*)}{\partial\mathcal{L}} + \mu(\mathcal{L}^{k+1}+\mathcal{E}^k-\mathcal{X}+\dfrac{\mathcal{Y}^k}{\mu^k}), \\ 0 = \dfrac{\partial(\lambda\|\mathcal{E}\|_1)}{\partial\mathcal{E}} + \mu(\mathcal{L}^{k+1}+\mathcal{E}^{k+1}-\mathcal{X}+\dfrac{\mathcal{Y}^k}{\mu^k}). \end{cases}$$

Substitute and scale using the following formula,

$$\mathcal{Y}^{k+1} = \mathcal{Y}^k + \mu^k \times (\mathcal{L}^{k+1}+\mathcal{E}^{k+1}-\mathcal{X}),$$

we have

$$\mu^k(\mathcal{L}^{k+1}-\mathcal{L}^k) = -\frac{\partial(\|\mathcal{L}\|_*)}{\partial\mathcal{L}} - \mu^k(\frac{\mathcal{Y}^k}{\mu^k} + \frac{\mathcal{Y}^k-\mathcal{Y}^{k-1}}{\mu^{k-1}}),$$
$$\mu^k(\mathcal{E}^{k+1}-\mathcal{E}^k) = -\frac{\partial(\lambda\|\mathcal{E}\|_1)}{\partial\mathcal{L}} - \mu^k\frac{\mathcal{Y}^k}{\mu^{k-1}}.$$

Where, we define V_1 and V_2,

$$\begin{cases} V_1^{k+1} := -\dfrac{\partial(\|\mathcal{L}\|_*)}{\partial\mathcal{L}} - \mu^k(\dfrac{\mathcal{Y}^k}{\mu^k} + \dfrac{\mathcal{Y}^k-\mathcal{Y}^{k-1}}{\mu^{k-1}}), \\ V_2^{k+1} := -\dfrac{\partial(\lambda\|\mathcal{E}\|_1)}{\partial\mathcal{L}} - \mu^k\dfrac{\mathcal{Y}^k}{\mu^{k-1}}. \end{cases}$$

Then,

$$\omega_1^{k+1} := V_1^{k+1} + \nabla_{\mathcal{L}}P_{\mathcal{L}}(\mathcal{L}^{k+1}) \in \partial P(\mathcal{L}^{k+1}),$$
$$\omega_2^{k+1} := V_2^{k+1} + \nabla_{\mathcal{E}}P_{\mathcal{E}}(\mathcal{E}^{k+1}) \in \partial P(\mathcal{E}^{k+1}).$$

There exists $\rho_3 > 0$ and $\rho_4 > 0$, such that

$$\left\|\omega_1^{k+1}\right\|_F \le \rho_3\left\|\mathcal{L}^{k+1}-\mathcal{L}^k\right\|_F, \quad \omega_1^{k+1} \in \partial P(\mathcal{L}^{k+1}),$$
$$\left\|\omega_2^{k+1}\right\|_F \le \rho_4\left\|\mathcal{E}^{k+1}-\mathcal{E}^k\right\|_F, \quad \omega_2^{k+1} \in \partial P(\mathcal{E}^{k+1}).$$

The proof of the relative error property lemma has been completed. □

Lemma 6 (Continuity): Assuming $infP > -\infty$, show that exists two subsequences $\left\{\mathcal{L}^{k_t}\right\}_{t\in N}$ and $\left\{\mathcal{E}^{k_t}\right\}_{t\in N}$, let $t \to +\infty$, such that

$$\mathcal{L}^{k_t} \longrightarrow \bar{\mathcal{L}}, \quad P(\mathcal{L}^{k_t}) \longrightarrow P(\bar{\mathcal{L}}),$$
$$\mathcal{E}^{k_t} \longrightarrow \bar{\mathcal{E}}, \quad P(\mathcal{E}^{k_t}) \longrightarrow P(\bar{\mathcal{E}}).$$

Proof Assuming there are two convergent subsequences $\mathcal{L}^{k_t}$ and $\mathcal{L}^{k_t-1}$ that satisfy the sufficient decrease property of Lemma 3, $\mathcal{L}^{k_t} \to \bar{\mathcal{L}}$, $\mathcal{L}^{k_t-1} \to \bar{\mathcal{K}}$, for positive parameter ρ_5, we have

$$\rho_5\left\|\mathcal{L}^0-\mathcal{L}^1\right\|_F^2 \le P(\mathcal{L}^0) - P(\mathcal{L}^1),$$
$$\rho_5\left\|\mathcal{L}^1-\mathcal{L}^2\right\|_F^2 \le P(\mathcal{L}^1) - P(\mathcal{L}^2),$$
$$\vdots$$
$$\rho_5\left\|\mathcal{L}^{t-1}-\mathcal{L}^t\right\|_F^2 \le P(\mathcal{L}^{t-1}) - P(\mathcal{L}^t).$$

By summing the above equations, we can obtain

$$\rho_5\sum_{k=0}^{t}\left\|\mathcal{L}^k-\mathcal{L}^{k+1}\right\|_F^2 \le P(\mathcal{L}^0) - P(\mathcal{L}^{t+1}).$$

According to the conditions, it can be inferred that, $\left\{P(\mathcal{L}^t)\right\}$ is decreasing and $infP > -\infty$, there exist $\bar{P}$. Let $t \to +\infty$, then $P(\mathcal{L}^t) \longrightarrow P(\bar{\mathcal{L}})$, and we have

$$\sum_{k=0}^{t}\left\|\mathcal{L}^k-\mathcal{L}^{k+1}\right\|_F^2 \le P(\mathcal{L}^0) - \bar{P} < +\infty.$$

we can infer that

$$\lim\left\|\mathcal{L}^k-\mathcal{L}^{k-1}\right\|_F = 0.$$

And we have $\lim\left\|\mathcal{L}^{k_{t+1}}-\mathcal{L}^{k_t}\right\|_F = 0$.

Based on the assumed conditions, it can show that $\bar{\mathcal{L}} = \bar{\mathcal{K}}$.

We denote subsequences $\bar{\mathcal{L}} = (\bar{\mathcal{L}}_1, \bar{\mathcal{L}}_2, \cdots, \bar{\mathcal{L}}_N)$, then for all $\mathcal{L}_i$, we have

$$\left\|\mathcal{L}_i^{k+1}\right\|_* + \lambda\left\|\mathcal{E}^k\right\|_1 + \frac{\mu^k}{2}\left\|\mathcal{L}_i^{k+1}+\mathcal{E}^k-\mathcal{X}+\frac{\mathcal{Y}^k}{\mu^k}\right\|_F^2 - \frac{\mu^k}{2}\left\|\frac{\mathcal{Y}^k}{\mu^k}\right\|_F^2$$
$$\le \|\mathcal{L}_i\|_* + \lambda\left\|\mathcal{E}^k\right\|_1 + \frac{\mu^k}{2}\left\|\mathcal{L}_i+\mathcal{E}^k-\mathcal{X}+\frac{\mathcal{Y}^k}{\mu^k}\right\|_F^2 - \frac{\mu^k}{2}\left\|\frac{\mathcal{Y}^k}{\mu^k}\right\|_F^2.$$

Let $k = k_t - 1$, $\mathcal{L}_i = \bar{\mathcal{L}}_i$ and $t \to +\infty$, then

$$\mathcal{L}_i^{k+1} = \mathcal{L}_i^{k_t} = \bar{\mathcal{L}},$$
$$\mathcal{L}_i = \bar{\mathcal{L}}_i = \bar{\mathcal{K}}.$$

And we can see that $P(\mathcal{L}^{k_t}) \longrightarrow P(\bar{\mathcal{L}})$, $t \to +\infty$.

Similarly, there is a similar conclusion for $\mathcal{E}$: show that exists a subsequence $\left\{\mathcal{E}^{k_t}\right\}_{t\in N}$, let $t \to +\infty$, such that

$$\mathcal{E}^{k_t} \longrightarrow \bar{\mathcal{E}}, \quad P(\mathcal{E}^{k_t}) \longrightarrow P(\bar{\mathcal{E}}).$$

The proof of the continuity lemma has been completed. □

Now, we prove the Theorem 1.

Proof Lemma 3 establishes that the function $P(\mathcal{L},\mathcal{E},\mathcal{Y},\mu)$ satisfies the KŁ property, according to Lemma 4, Lemma 5 and Lemma 6, we prove the sequence $\left\{\mathcal{L}^k,\mathcal{E}^k\right\}$ satisfies the sufficient decrease, relative error and continuity properties.

Therefore, the sequence $\left\{\mathcal{L}^k,\mathcal{E}^k\right\}$ globally converges to a critical point of equation (3.2). The proof of theorem 1 is completed. □

Supplementary figures

The Figure 4-11 are the supplementary figures for the main manuscripts.

References

1. Wold, S., Esbensen, K., and Geladi, P. Principal component analysis. *Chemometrics and intelligent laboratory systems*, **2**(1-3), 37–52, 1987.
2. Zhao, X.-L., Yang, J.-H., Ma, T.-H., Jiang, T.-X., Ng, M. K., and Huang, T.-Z. Tensor completion via complementary global, local, and nonlocal priors. *IEEE Transactions on Image Processing*, **31**, 984–999, 2021.
3. Bao, C., Ji, H., Quan, Y., and Shen, Z. Dictionary learning for sparse coding: Algorithms and convergence analysis. *IEEE transactions on pattern analysis and machine intelligence*, **38**(7), 1356–1369, 2015.
4. Attouch, H., Bolte, J., and Svaiter, B. F. Convergence of descent methods for semi-algebraic and tame problems: proximal algorithms, forward–backward splitting, and regularized gauss–seidel methods. *Mathematical Programming*, **137**(1), 91–129, 2013.
5. Bolte, J., Sabach, S., and Teboulle, M. Proximal alternating linearized minimization for nonconvex and nonsmooth problems. *Mathematical Programming*, **146**(1), 459–494, 2014.
6. Ponnapalli, S. P., Saunders, M. A., Van Loan, C. F., and Alter, O. A higher-order generalized singular value decomposition for comparison of global mrna expression from multiple organisms. *PloS one*, **6**(12), e28072, 2011.

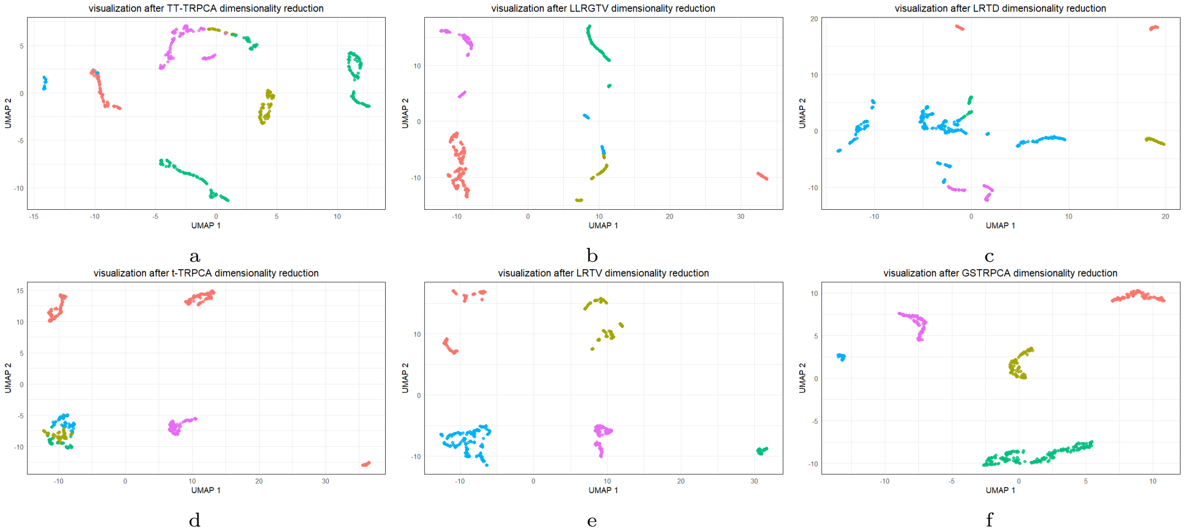

Fig. 4. Visualization of cell clusters after dimension reduction for different methods on Sim1. (a) TT-TRPCA, (b) LRTD, (c) LLRGTV, (d) t-TRPCA, (e) LRTV and (f) GSTRPCA

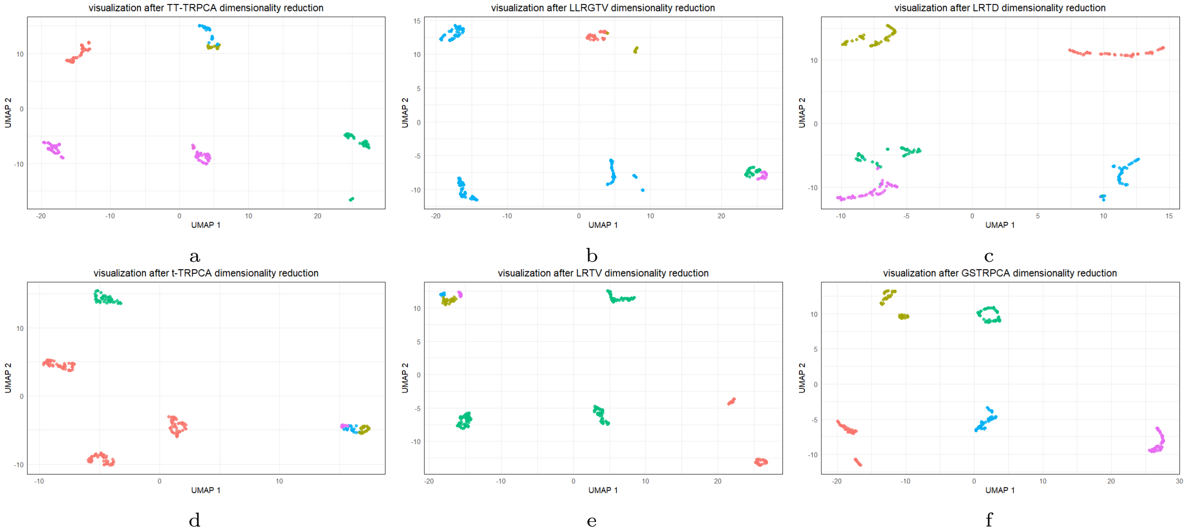

Fig. 5. Visualization of cell clusters after dimension reduction for different methods on Sim2. (a) TT-TRPCA, (b) LRTD, (c) LLRGTV, (d) t-TRPCA, (e) LRTV and (f) GSTRPCA

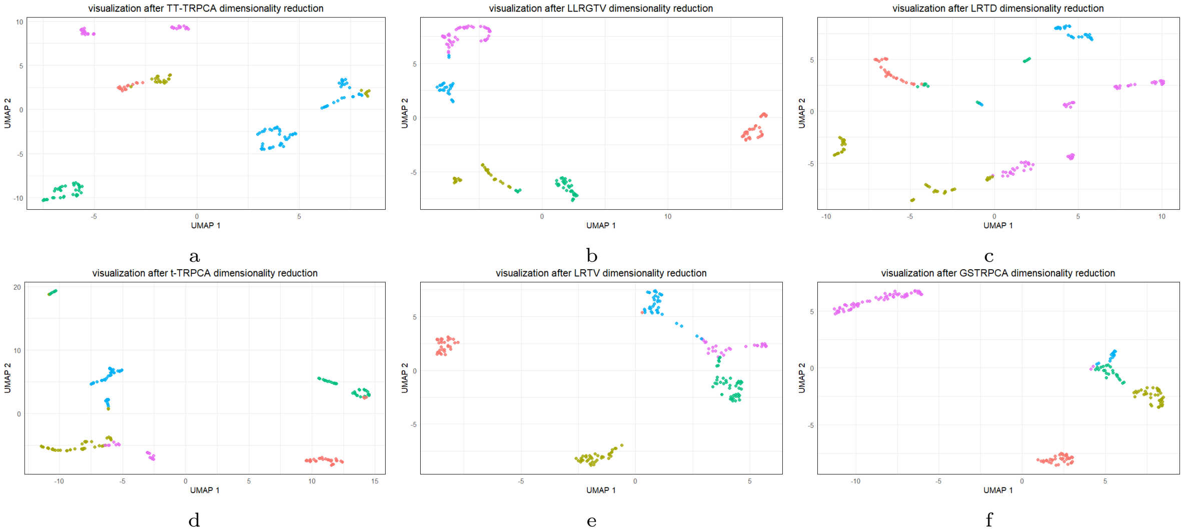

Fig. 6. Visualization of cell clusters after dimension reduction for different methods on SCGEM. (a) TT-TRPCA, (b) LRTD, (c) LLRGTV, (d) t-TRPCA, (e) LRTV and (f) GSTRPCA

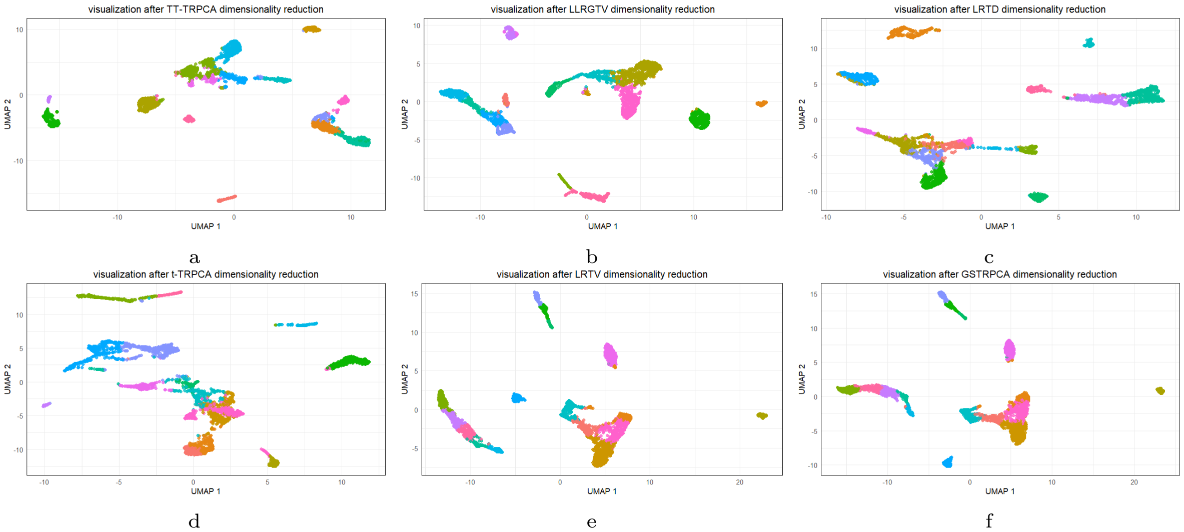

Fig. 7. Visualization of cell clusters after dimension reduction for different methods on Specter. (a) TT-TRPCA, (b) LRTD, (c) LLRGTV, (d) t-TRPCA, (e) LRTV and (f) GSTRPCA

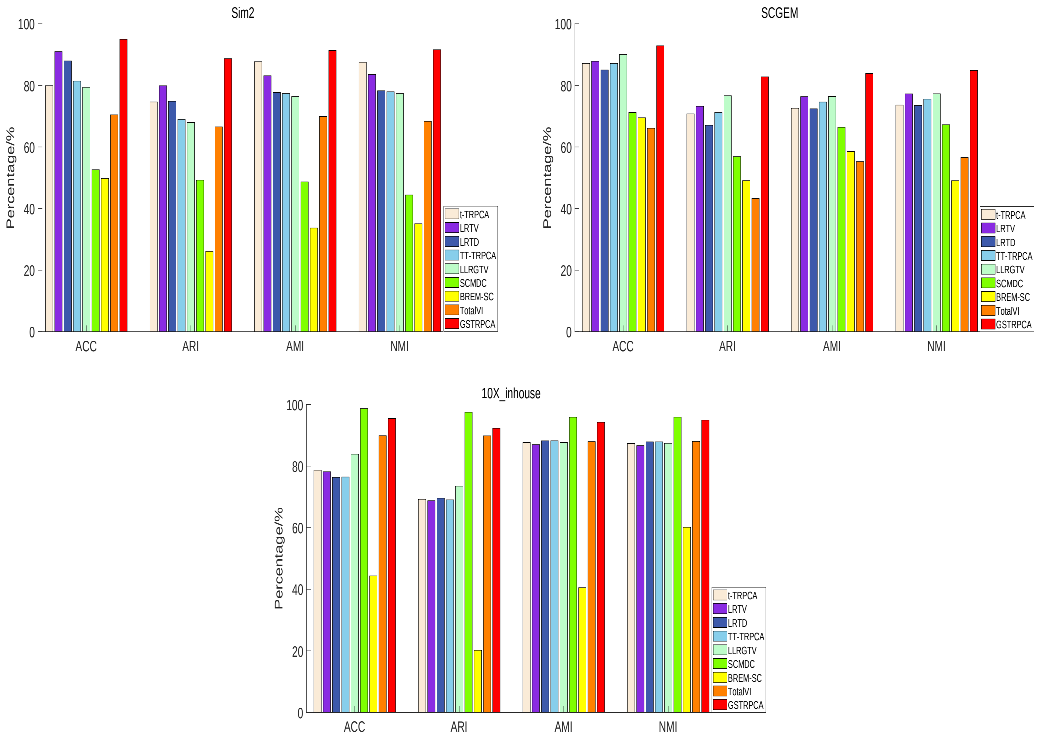

Fig. 8. Comparison of evaluation metrics performance of GSTRPCA and competing methods on five datasets.

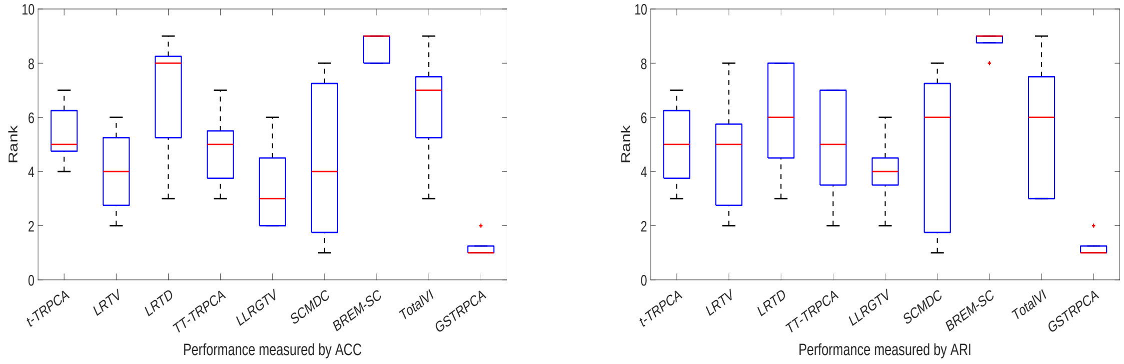

Fig. 9. Boxplots of GSTRPCA and other competing algorithms on five datasets.

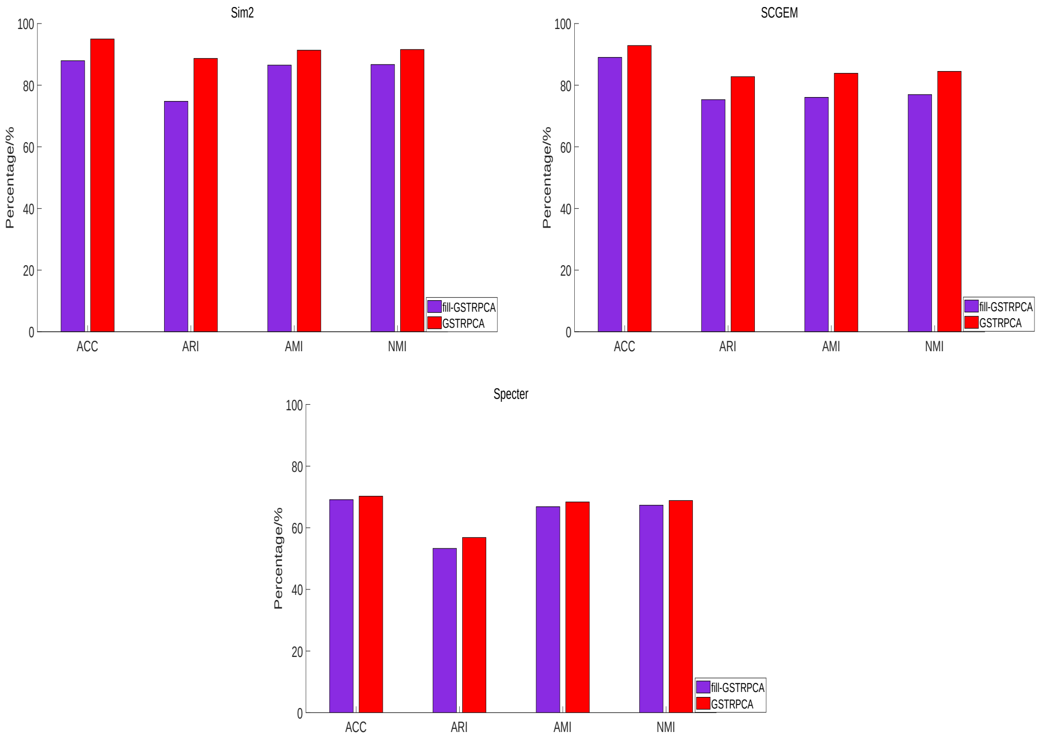

Fig. 10. Comparison of clustering performance between the GSTRPCA decomposition of irregular tensor and fill-GSTRPCA decomposition of regular tensor on datasets Sim2, SCGEM and Specter.

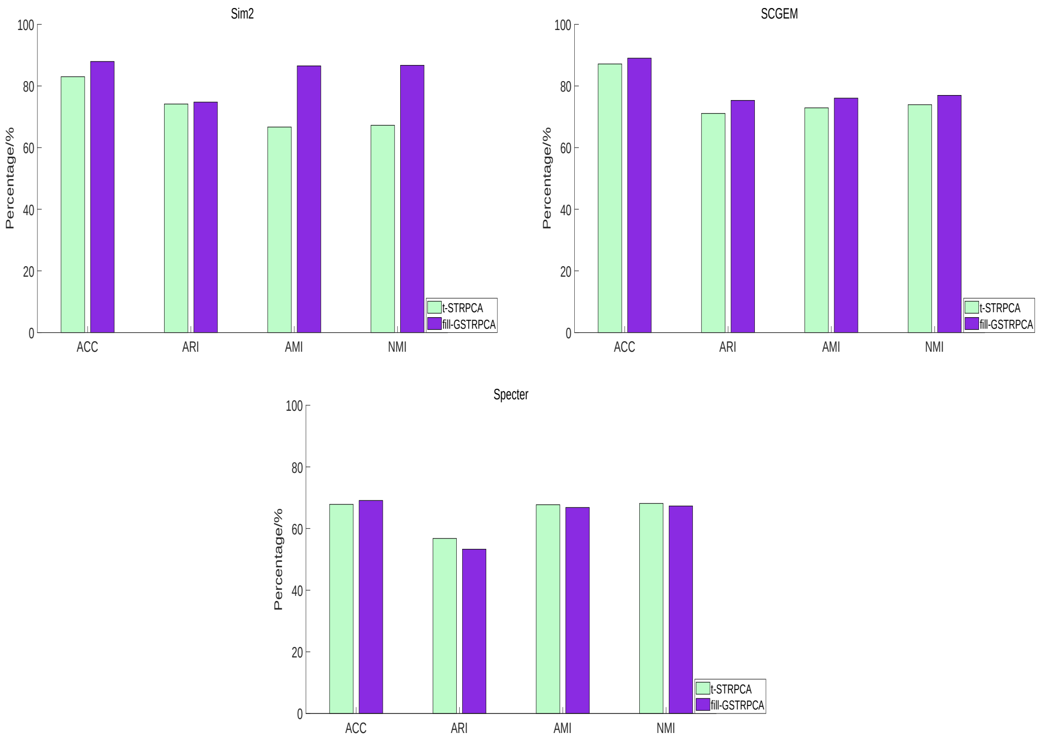

Fig. 11. Performance comparison of fill-GSTRPCA and t-STRPCA decomposition methods on datasets Sim2, SCGEM and Specter.
